# Climate-smart cowpea adoption and welfare effects of comprehensive agricultural training programs

**DOI:** 10.1016/j.techsoc.2020.101468

**Published:** 2021-02

**Authors:** Edward Martey, Prince M. Etwire, Jonathan Mockshell

**Affiliations:** a Socio-economic section, CSIR-Savanna Agricultural Research Institute, P.O. Box TL 52, Tamale, Ghana; b International Center for Tropical Agriculture (CIAT), Apartado Aereo 6713, Cali, Colombia

**Keywords:** Comprehensive training programs, Climate-smart agriculture, Endogenous switching regression, Adoption, Welfare effects

## Abstract

Agricultural training programs remain one of the primary mechanisms for disseminating modern and climate-smart technologies with the aim to improve the welfare outcomes of smallholder farmers. With persistent low agricultural productivity in Sub Saharan Africa (SSA), the content, effectiveness, and mode of delivery of training programs remain a debate. In this paper, we examine the adoption, productivity, and income effects of participating in a novel comprehensive agricultural training program (CATP) involving cowpea farmers in northern Ghana by using the endogenous switching regression (ESR) model. The CATP requires farmers to complete a set of modules on good agronomic practices to gain informal certification. The results indicate that participating in the CATP increases the adoption of climate-smart cowpea varieties, productivity, and cowpea income by 75, 15, and 24% points, respectively, compared to their mean levels. These positive welfare effects of participating in the CATPs confirm the need to increase capacity-enhancing activities in agricultural development projects, and design mechanisms to eliminate barriers to participation among rural farm households.

## Highlights

• Comprehensive agricultural training programs (CATPs) facilitate the dissemination of climate-smart technologies.
• Limited evidence of comprehensive agricultural training programs among practitioners.
• Participation in CATP is significantly enhanced by socio-demographic and institutional factors.
• Participation in CATP increases the adoption of climate-smart cowpea varieties, yield, and cowpea income.
• The gains in welfare outcomes can be consolidated through agricultural development projects.

## 1 Introduction

In developing countries, most households depend on the agricultural sector for livelihood support. Yet, persistent low agricultural productivity and limited transformation of the food system are significant hurdles to improving welfare [1,2]. Several past and current development interventions prioritize increasing agricultural productivity as a viable pathway to improving farmers’ welfare. These interventions include increasing access to technologies such as fertilizer, improved seed varieties, and technical knowledge [3,4]. Agricultural training programs that boost human capital remain one of the mechanisms for improving the knowledge of farmers on emerging technological innovations [5,6]. However, there is limited consensus on the effectiveness of existing training approaches. For example, whereas the training and visit agricultural extension approach is often criticized as a top-down approach, there is limited agreement on the effectiveness of other models, such as farmer field schools (FFS) [7]. While some empirical analysis has found positive impacts of FFS on yield and income, other studies suggest the opposite [[7], [8], [9]]. This study aims at contributing to evidence-based research on the impact of a comprehensive agricultural training programs (CATPs) on the adoption of climate-smart cowpea varieties, yield, and income.

Improving the technical skills of farmers using appropriate training methods is particularly crucial in Sub Saharan Africa (SSA) where agricultural productivity and farm incomes are low (). Despite the growing body of evidence, the number of capacity building interventions in Africa outweighs the number of studies that assess the effects of such interventions on the welfare of recipients [8]. Most studies have focused on FFS, farmer-to-farmer extension, or the use of model farmers [8,10]. In this study, we argue that despite the importance of these approaches, farmer participation may not necessarily translate into capacity building. Farmers’ participation in agricultural programs may be active or passive depending on several factors, such as education level, income, and social networks [4,11]. Moreover, it is possible that farmers do not complete all of the training module (e.g., modules from seed selection to postharvest). In such circumstances, analyzing the impact of training on welfare may not be an accurate reflection given that full and partial participants may have different outcomes when compared to non-participants.

A multidisciplinary research team from the Savanna Agricultural Research Institute (SARI) of Ghana's Council for Scientific and Industrial Research (CSIR) initiated a community training school in 2017. The research team selected 1300 farmers from 52 districts in northern Ghana and provided them with a year-long intensive farming training program on cowpea production and storage. The specific training modules included identification of quality seed, storage of cowpea seed, use of inoculant, planting (timing and method of planting, namely, row planting and spacing), effective weed management practices, pest management practices, soil fertility management methods, fertilizer application (method, quantity, and timing), suitable harvesting methods (timing and methods), and postharvest management practices (proper storage methods, such as using hermetic or triple-layered bags). The research team in collaboration with local extension officers, combined both practical field demonstration and formal adult learning techniques to train selected farmers. Local extension officers act as a backstop and are a sustainable source of agricultural information. Strict protocols were discussed and adopted by the research team and participating farmers to ensure compliance and active participation. In addition, routine monitoring of the training program was carried out by local extension officers.

Some earlier studies of the impacts on training programs did not explicitly address potential biases associated with non-random program placement and the selection of farmers [9]. Such studies do not adequately control for observed and unobserved differences that exist between trained and untrained farmers thereby making it difficult to draw conclusive statements given that communities, for example, may be chosen to participate in training programs because of fertile lands or conduciveness for crop production. Similarly, farmers who opt to participate in training programs may be those who are naturally more productive. Hence, a comparison of participants against nonparticipants may not provide a fair basis for constructing counterfactuals. A recent study by Ref. [10] using panel data showed that farmer-to-farmer training increased adoption of improved rice and yields among early trainees while subsequently trained farmers caught up belatedly. This study complements and expands existing empirical analyses by exploring how comprehensive agricultural training provided by a research institution impacts climate-smart cowpea adoption, productivity, and income. To the best of our knowledge, there is no other empirical study in Ghana that evaluates the impact of a novel training program on the welfare of rural households.

This analysis evaluates the impact of a CATP on climate-smart cowpea variety adoption, yield, and cowpea income. In the absence of baseline information, the paper uses the endogenous switching regression (ESR) model to deal with both selection bias and unobservable factors that can lead to biased results. Subsequently, we estimate propensity score matching and inverse probability weighted regression adjustment as robustness checks. Our empirical estimates show that completing cowpea training modules increases adoption of climate-smart cowpea varieties, cowpea yield, and cowpea income by 75, 15 and 24% points, respectively, compared to their mean levels.

The rest of the paper proceeds as follows. In the next section, we provide a description of our data generation process and the variables in the model. Section 3 consists of our conceptual framework and estimation strategies as well as a description of our main method of analysis, the endogenous switching regression model. We present and discuss our empirical estimates in Section 4 before presenting the conclusions and policy implications of our study in Section 5.

## 2 Study area, sampling technique, and data

**Fig. 1:**
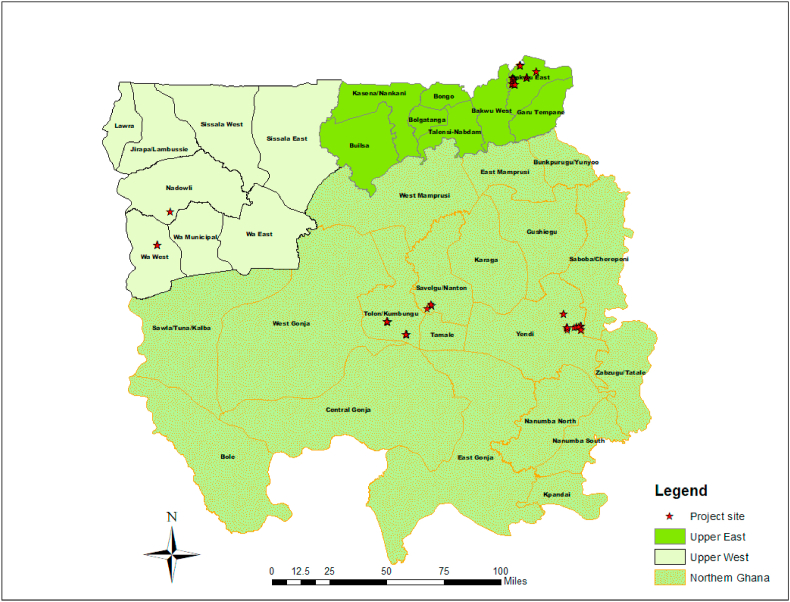
Study area indicating the data collection sites in northern Ghana.

Fig. 1 presents the map of the study area showing the location (districts) of the farmers interviewed. This study uses farm household survey data collected in 2019 for the 2018/2019 cropping season from 320 cowpea farmers in seven major cowpea growing districts (Tolon, Savelugu, Yendi, Bawku Municipal, Binduri, Wa West, and Nadowli) in northern Ghana.

This study forms part of a more extensive study commissioned by ICRISAT and IITA under the project “Accelerated Varietal Improvement and Seed Delivery of Legumes and Cereals in Africa (AVISA).” The AVISA project aims at refocusing its work to improve the Consortium of International Agricultural Research Centers (CGIAR) and crucial National Agricultural Research Systems (NARS) breeding and seed delivery systems. The AVISA initiative targets the most critical dryland cereals (sorghum and pearl millet) and legume crops (groundnut, common bean, and cowpea) on the African continent within the Bill and Melinda Gates Foundation (BMGF) focused geographies (Burkina Faso, Ghana, Mali, Nigeria, Tanzania and Uganda) where these crops are important. Capacity building is a significant component of the AVISA intervention. area showing the location (districts) of the farmers interviewed.

The sampling^1^ procedure followed a multi-stage sampling technique to select 320 farmers in the Guinea and Sudan Savannah agro-ecological zones consisting of the Northern, Upper East, and Upper West regions of northern Ghana. In the first stage, seven districts were purposively selected from these regions based on the quantity of cowpea produced, as well as the accessibility and presence of active Farmer-based Organizations (FBOs). In the second stage, 20 communities were purposively selected from the Northern Region and 10 communities each from the Upper East and Upper West regions from a list of cowpea-producing communities in each of the selected districts based on the volume of cowpea production. Within the selected communities, eight cowpea producers were randomly selected from a list of cowpea producers. In all, 320 cowpea producers were selected from 40 communities within seven districts (Table 1). Fig. 1 presents the map of the study area showing the location (districts) of the farmers interviewed.

**Table 1:** Distribution of sampled cowpea farmers by region.

Region	Number of District	Number of Communities	Number of Households
Northern	3	20	160
Upper West	2	10	80
Upper East	2	10	80
Total	7	40	320

A power test^2^ was conducted on the study's sample. Given the intended power, we can derive the required sample size; and given the intended sample size, we can derive the resulting power. Following [12]: *n* = (*N*/1+*N*e^2^), where *N* denotes the total population of farmers in northern Ghana, i.e., 4,228,116 farmers consisting of 2,479,461 from the Northern Region, 1,046,545 from the Upper East Region, and 702,110 from the Upper West Region [13]; *e* denotes the margin of error of the sample, namely, 0.05 (95% confidence level), and *n* denotes the sample size. The derived margin of error of the sample is 6% when the sample is 320 (implying a 94% confidence level). The result implies that we are 94% confident in the results obtained from the sample used for this study. Therefore, the sample is representative, and the results can be generalized for the population of farmers in the major cowpea-producing areas of northern Ghana.

The process of data collection commenced with pre-testing of the survey instrument with feedback from the field interview used to refine the survey instrument. Enumerators were recruited, trained, and deployed to communities to conduct a household survey. The data used in this study contain detailed household demographic and socio-economic characteristics, such as sex, education, age, marital status, household size, nativity, and residential status. Farm characteristics include farming experience, farm size, type of crops cultivated, area owned and area under cultivation (including male and female-owned fields), and common fields. Social capital-related variables, namely, membership in a FBO, village savings and loan association (VSLA), or cooperatives, as well as access to a Member of Parliament or politician. Data on access to institutional and social amenities, such as access to agricultural extension services, district assembly, district capital, input factor markets, and output markets were also collected. Lastly, the survey captured data on participation in cowpea training.

The mean and standard deviations of the variables used in the regression analysis and their definitions are presented in Table 2. Our choice of variables is based on the existing literature [3,4,7,10,11,14,and15]]. The study captures the welfare of the household in terms of yield, measured in kilograms per hectare, and cowpea income, expressed in Ghana cedi (GHS^3^). Participation in cowpea training is expected to build human capital that will translate into adoption of climate-smart cowpea varieties, higher yields, and therefore an increase in household crop income. The average adoption, yield, and cowpea income are 67%, 847.46 kg/ha and GHS1486.31 (US$277), respectively. About 34% of sampled households participated in the cowpea training. The average farm household head is relatively young (44 years) belonging to the economically active age group and can therefore be expected to work for the next two decades. On average, 59% of farm households are headed by males. Household heads have an average of seven years of formal education and 20 years of farming experience. Household size is relatively large with an average of nine household members. With respect to social capital, about 19%, 53%, and 46% of farmers are members of a cooperative, FBO, and VSLA, respectively. The area under cowpea cultivation for the sampled farm household is 1.18 ha and farmers travel an average distance of 3.85 km to access extension information. About 25% of farm households have access to a Member of Parliament or politician which proxies for access to both agricultural and other social safety programs promoted by the Government of Ghana with financial support from development partners.

**Table 2:** Variable name, description and summary statistics.

Variable	Definition	Mean	SD
**Dependent variable**			
Adoption	Adoption of improved cowpea varieties (1 = adoption)	0.67	0.47
Yield	Cowpea yield (kg/ha)	2118.65	1488.93
Crop Income	Cowpea income (GHS)	1486.31	1227.99
**Treatment variable**			
Training	Participated in cowpea training (1 = yes)	0.34	0.47
**Explanatory variables**			
Age	Age of household head (number of years)	43.68	12.40
Sex	Sex of household head (1 = male)	0.59	0.49
Education	Number of years of formal education	6.98	4.68
Size	Household size	9.38	4.51
Experience	Years of farming experience	20.19	11.80
Cooperative	Member of cooperative (1 = yes)	0.19	0.39
FBO	Member of a farmer-based organization (1 = yes)	0.53	0.50
VSLA	Member of a village savings and loan association (1 = yes)	0.46	0.50
Cowpea area	Area under cowpea (hectares)	1.18	0.76
Member of Parliament	Access to Member of Parliament or politician (1 = yes)	0.25	0.43
Extension	Distance to nearest extension service (km)	3.85	7.85
**District level controls**			
Tolon district	Household is located in Tolon district (1 = yes)	0.15	0.36
Savelugu district	Household is located in Savelugu district (1 = yes)	0.18	0.38
Yendi district	Household is located in Yendi district (1 = yes)	0.18	0.38
Bawku Municipal	Household is located in Bawku district (1 = yes)	0.09	0.29
Binduri district	Household is located in Binduri district (1 = yes)	0.16	0.37
Wa West	Household is located in Wa West district (1 = yes)	0.13	0.33
Nadowli district	Household is located in Nadowli district (1 = yes)	0.13	0.33

Note: Exchange rate: US$1 = GHS5.37 (Bank of Ghana, 2019).

## 3 Methodology

### 3.1 Conceptual framework and estimation strategies

Participation in a CATP and its effect on the adoption of improved cowpea varieties, yield, and cowpea income are considered to be a stepwise decision-making process. The decision to participate in a CATP is a behavioral response and thus modelled as a random utility function, where a set of alternatives and constraints are faced by the decision-maker [14,15]. A risk-neutral utility maximizing cowpea grower, i decides to participate in a CATP if the utility derived from training ($U_{iA}$) exceeds that from otherwise ($U_{iN}$), such that the difference ($D_{i}^{*}$) between the two states is given by:

(1) $$D_{i}^{*}=U_{iA}-U_{iN}>0$$

where $D_{i}^{*}$ is a latent variable that captures the expected benefit from the choice of participation in CATP concerning non-participation and can therefore be expressed as a function of observable components in the latent variable model below:

(2) $$D_{i}^{*}=Z_{i}\alpha +\varepsilon_{i}>0\;where\;D_{i}=\left\{ \begin{array}{l} 1\;if\;D_{i}^{*}>0\\ 0\;otherwise, \end{array}\right.$$

where D is a binary decision variable that equals to one if cowpea farmer chooses to participate in a CATP and equals zero otherwise; Z is a vector of household demographics, socio-economic, and farm-level characteristics; α is a vector of parameters to be estimated; and $\varepsilon_{i}$ is a random error term. In this paper, we define a cowpea trained farmer as any cowpea farmer who has completed all modules of the CATP.

Participation in a CATP is expected to influence the adoption of improved cowpea varieties, yield, and cowpea income. The outcome variable is expressed as a function of a vector, X, of variables and an endogenous adoption variable (D), such that:

(3) $$Y_{l}=\omega X_{i}+\delta D_{i}+\mu_{i}$$

where $Y_{i}$ represents the outcome variables (adoption of improved cowpea varieties, yield and cowpea income), D is the participation variable defined previously, ω and δ are parameters to be estimated, and μ is an error term. Since farmers were not randomly assigned, estimates of δ will be biased. Trained farmers may be systematically different from non-trained farmers, which may bias the actual effect of training on the outcome variables [16]. Estimating equation (3) with ordinary least squares (OLS) produces biased estimates. Due to this shortcoming, several methods have been proposed for non-experimental data including propensity score matching (PSM), inverse probability weighted regression adjustment (IPWRA), instrumental variables, and endogenous switching regression (ESR) models. To correct this biased estimate, we employ an ESR model to address the endogeneity in participation in cowpea training due to self-selection. Nonetheless, we use both the PSM and IPWRA as robustness checks. Details of the empirical frameworks for PSM and IPWRA can be found in Ref. [[3], [17]], respectively.

### 3.2 Endogenous switching regression (ESR)

Several studies have employed the ESR model [[18], [19], [20], [21], [22]]. Following [18], the ESR model consists of separate outcome equations for trained and non-trained farmers conditional on the choice of cowpea training decisions, namely:

(4a) $$\text{Regime }1\;\left(\text{Trained in CATP}\right)\text{ :}\;Y_{1i}=\beta_{1}X_{1i}+e_{1i}\;if\;D_{i}=1$$

(4b) $$\text{Regime }2\;\left(\text{Untrained in CATP}\right)\text{ :}\;Y_{2i}=\beta_{2}X_{2i}+e_{2i}\;if\;D_{i}=0$$

where $Y_{1i}$ and $Y_{2i}$ are the outcome variables for trained and non-trained, $X_{1i}$ and $X_{2i}$ are household and farm level characteristics; $\beta_{1}$ and $\beta_{2}$ are parameters to be estimated for trained and non-trained farmers regimes, respectively; and $e_{1}$ and $e_{2}$ are random disturbance terms.

For the ESR model to be identified, the variables in the choice model (equation (2)) must contain an exclusion restriction variable [23]. Following the example of [[24], [25], [26]], we use access to a member of parliament or whether a political figure resides in the constituency, district, or community as an instrument. Members of Parliament or politician can dictate the placement of developmental projects in their areas of jurisdiction and farmers who are related to or have access to such political figures are more likely to be included in agricultural development projects. This variable is likely to be correlated with the choice of participation in a CATP but is unlikely to have any direct effect on the outcome variables. The admissibility of the instrument is established by performing a simple falsification test^4^ following the approach of [22].

Following the specification of [3], the error terms ($\varepsilon_{i}$, $e_{1i}$, and $e_{2i}$) are assumed to have a trivariate normal distribution with mean vector zero and covariance matrix:

(5) $$cov\left(\varepsilon ,\;e_{1},\;e_{2}\right)=\left(\begin{array}{l} \begin{array}{l} \begin{array}{lll} \sigma_{\varepsilon }^{2} & \sigma_{e_{1}\varepsilon } & \sigma_{e_{2}\varepsilon } \end{array}\\ \begin{array}{lll} \sigma_{e_{1}\varepsilon } & \sigma_{e_{1}}^{2} & \sigma_{e_{1}e_{2}} \end{array} \end{array}\\ \begin{array}{lll} \sigma_{e_{2}\varepsilon } & \sigma_{e_{1}e_{2}} & \sigma_{e_{2}}^{2} \end{array} \end{array}\right)$$

where $\sigma_{\varepsilon }^{2}$ = var (ε), which is assumed to be 1 since γ is only estimable up to a scale factor [27], $\sigma_{e_{1}}^{2}$ = var ($e_{1}$), $\sigma_{e_{2}}^{2}$ = var($e_{2}$), $\sigma_{e_{1}\varepsilon }$ = cov ($e_{1},\varepsilon$), and $\sigma_{e_{2}\varepsilon }$ = cov ($e_{2},\varepsilon$). The covariance between $e_{1}$ and $e_{2}$ is not defined since $Y_{1}$ and $Y_{2}$ are never observed simultaneously [27]. The expected values of the error terms $e_{1}$ and $e_{2}$ can be expressed as:

(6) $$E\left(e_{1}|D_{i}=1\right)=\sigma_{e_{1}\varepsilon }\lambda_{1}\;and$$

(7) $$E\left(e_{2}|D_{i}=0\right)=\sigma_{e_{2}\varepsilon }\lambda_{2}$$

where $\lambda_{1}$ and $\lambda_{2}$ are the inverse mils ratio (IMR) calculated from the selection equations (2) and included in the outcome equations (4a) and (4b) to correct for selection bias in a two-step estimation procedure known as the endogenous switching treatment regression model [27]. The new outcome equations for the two regimes can be specified as:

(8) $$Y_{1i}=\beta_{1}X_{1i}+\sigma_{e_{1}\varepsilon }\lambda_{1}+\omega_{1}\;if\;D_{i}=1\;and$$

(9) $$Y_{2i}=\beta_{2}X_{2i}+\sigma_{e_{2}\varepsilon }\lambda_{0}+\omega_{2}\;if\;D_{i}=0$$

If $\sigma_{e_{1}\varepsilon }$ and $\sigma_{e_{2}\varepsilon }$ in equations (8) and (9) are statistically significant, then there is endogenous switching; otherwise, there is exogenous switching. The full information maximum likelihood (FIML) estimation approach is used by the study to estimate the selection and outcome equations simultaneously. This method is more efficient than the two-step procedure [29,30]. The coefficients from the ESR model can be used to derive the average treatment effect on the treated (ATT) and the average treatment effect on the untreated (ATU) by comparing the expected values of the outcomes of participation and non-participation in actual and counterfactual scenarios. The ATT, ATU, base heterogeneity (BH), and transitional heterogeneity (TH) are calculated following [22,23]. Detailed representations of the calculations are reported in Ref. [3].

### 3.3 Non-parametric regression and stochastic dominance analysis

The study employs a non-parametric local polynomial regression to establish the existing relationship between the outcome variables and the CATP. We also use the stochastic dominance analysis (SDA) to compare the cumulative distributions of adoption, cowpea yields, and cowpea income among participants and non-participants of the CATP. This method focuses on the distribution of the mean and the variance of the economic measures (adoption, yield, and cowpea income) for households matched based on their propensity score of participation within the region of common support. This paper used the first two orders of stochastic dominance to differentiate between participation and non-participation in cowpea training. Following the definitions by Ref. [31], the first-degree stochastic dominance (FSD) order states that if one cumulative distribution is to the left of another cumulative distribution for all levels of the outcome variable of interest, then participating households with the distribution to the right would stochastically dominate those to the left. The second stochastic dominance (SSD) order assumes that human beings are risk averse and prefer to avoid lower outcomes. Graphically, a technology would stochastically dominate in the analysis if the area under its cumulative probability curve is smaller at every outcome level compared to the curve of the alternative [31]. The test for first-order stochastic dominance is conducted using the nonparametric Kolmogorov–Smirnov test.

## 4 Results and discussion

### 4.1 Descriptive statistics

Table 3 reports the mean values of our outcome and explanatory variables by training status. The results indicate that trained farmers recorded significantly higher adoption (78% vs. 62%) of climate-smart cowpea varieties and obtained higher yields (888.13 kg/ha vs. 826.45 kg/ha) compared to non-trained farmers, although the difference in yields is not statistically significant. Trained farmers recorded statistically significantly higher cowpea income (US$307) than non-trained farmers (US$261). Concerning the explanatory variables, trained farmers were significantly distinguishable in terms of age, member of a FBO, member of a VLSA, area under cowpea cultivation, and location (Savelugu and Wa West districts). Trained household heads are on average four years older than non-trained household heads. About 15% and 11% more trained household heads belong to a FBO and VSLA, respectively, compared to non-trained household heads. The area under cowpea cultivation controlled by trained farmers is 0.41 ha more than non-trained farmers.

**Table 3:** Descriptive statistics by training status.

Variable	Full Sample	Trained (T)	Non-trained (N)	Difference (T-N)
**Dependent variable**				
Climate-smart variety (%)	0.67	0.78	0.62	0.16***
Yield (kg/ha)	847.46	888.13	826.45	61.68
Crop Income (GHS)	1486.31	1648.30	1402.63	245.67*
**Explanatory variables**				
Age	43.68	46.58	42.18	4.40***
Sex	0.59	0.58	0.60	−0.02
Education	6.98	7.06	6.93	0.13
Size	9.38	9.88	9.12	0.76
Experience	20.19	21.63	19.45	2.19
Cooperative	0.19	0.15	0.21	−0.06
FBO	0.53	0.63	0.48	0.15***
VSLA	0.46	0.53	0.42	0.11*
Cowpea area	1.18	1.45	1.04	0.41***
Parliament member	0.25	0.28	0.23	0.06
Extension	3.85	4.49	3.53	0.96
Tolon district	0.15	0.13	0.16	−0.03
Savelugu district	0.18	0.33	0.09	0.25***
Yendi district	0.18	0.17	0.18	−0.01
Bawku Municipal	0.09	0.08	0.09	−0.01
Binduri district	0.16	0.12	0.18	−0.06
Wa West	0.13	0.07	0.15	−0.08**
Nadowli district	0.13	0.09	0.14	0.05

Note: Exchange rate: US$1 = GHS5.37 (Bank of Ghana, 2019).

**Fig. 2:**
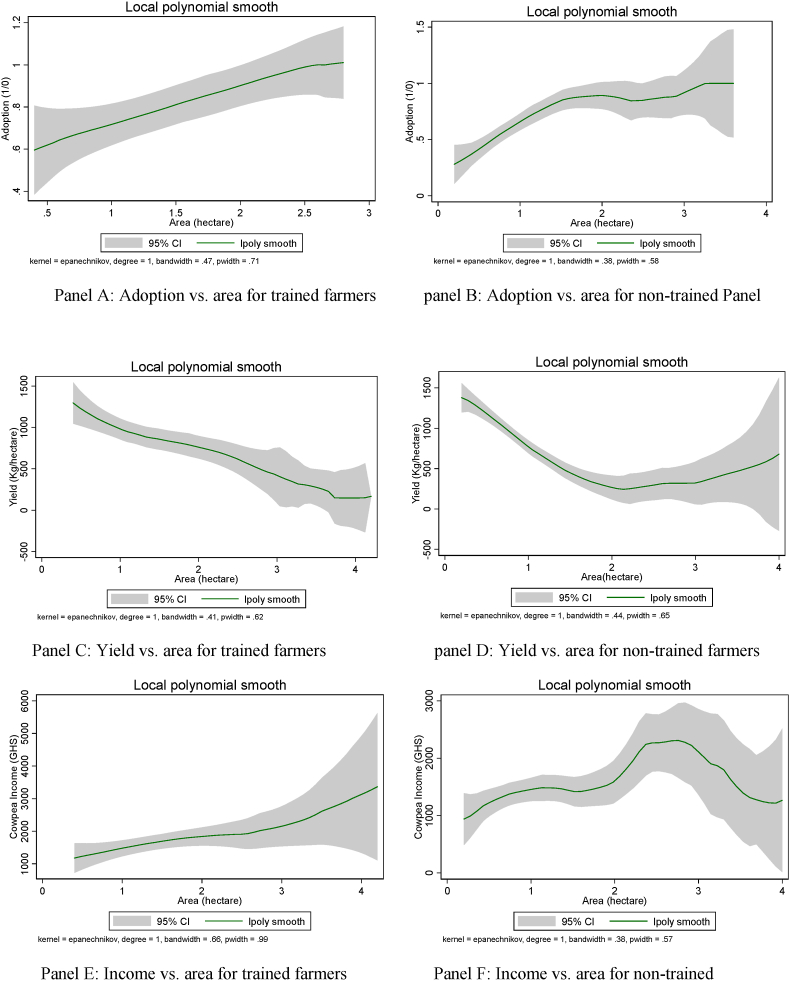
Local polynomial regressions for trained and non-trained cowpea farmers.

Fig. 2 shows the result of the non-parametric local polynomial regression that establishes a relationship between the following: adoption of climate-smart cowpea variety and area for trained farmers (panel A), adoption and area for non-trained farmers (panel B); cowpea yield and area for trained farmers (panel C), cowpea yield and area for non-trained farmers (panel D); cowpea income and area for trained farmers (panel E); and cowpea income and area for non-trained farmers (panel F). A smooth, positive trend is observed between adoption and area cultivated among trained farmers while a fluctuation is observed among non-trained farmers. The result highlights the importance of training in technology adoption. Consistent with the literature [3,32], we observed a negative relationship between cowpea yield and area under cultivation for trained and untrained farmers who cultivate less than 2 ha. This result indicates that farm households with relatively small landholdings are more likely to increase their productivity, especially in areas where mechanization support is absent. Panel E shows that among trained farmers, cowpea income and area exhibit a positive relationship for all areas of cowpea, but the observed pattern is different for non-trained cowpea farmers (panel F). The positive relationship only holds for non-trained farmers that have below 2.8 ha. Cowpea income decreases with farm size for non-trained farmers who own more than 2.8 ha under cowpea cultivation. No conclusive statement can be inferred given that other factors that might influence adoption, yield and cowpea income are not controlled in the local polynomial regression.

Table 4 shows the quintile distribution of adoption, cowpea yield, and cowpea income disaggregated by participation in the CATP. Trained farmers recorded a consistently higher rate of adoption of climate-smart cowpea varieties than non-trained farmers, with the majority of the trained farmers within the third and fourth quintiles of the adoption distribution. Apart from the second and fourth quintiles of yield distribution, trained farmers recorded higher yields than non-trained farmers. However, the fifth quintile of non-trained farm households did not record any yield indicating a share of zero. Similarly, trained farmers have a relatively higher income than non-trained farmers. The first quintile (poorest) of the sample farm households recorded 4% (trained) and 5% (non-trained) of the total cowpea income relative to the fifth quintile (richest) who recorded 46% of the total cowpea income. The poorest trained farmers recorded higher yields than the poorest non-trained farmers, but the poorest non-trained farmers received relatively higher cowpea income than the poorest trained farm households. The results may be attributed to differences in the market price received by the poorest non-trained farmers compared to the poorest trained farmers.

**Table 4:** Distributional summary statistics for adoption, yield and cowpea income.

Quintiles	First	Second	Third	Fourth	Fifth
	Quintile	Quintile	Quintile	Quintile	Quintile
**Adoption (1/0)**					
Trained	0.81	0.70	0.89	0.87	0.61
Share (%)	(20.88)	18.04	(22.94)	(22.42)	(15.72)
Non-trained	0.81	0.69	0.68	0.45	0.00
Share (%)	(30.80)	26.24	(25.86)	(17.11)	(0.00)
**Cowpea yield (kg/ha)**					
Trained	171.63	402.74	781.43	1261.98	1727.17
Share (%)	(3.95)	9.27	(17.98)	(29.04)	(39.75)
Non-trained	61.17	420.05	785.67	1486.70	0.00
Share (%)	(2.22)	15.25	(28.53)	(53.99)	(0.00)
**Cowpea income (GHS)**					
Trained	309.52	754.55	1208.07	1955.00	3396.73
Share (%)	(4.06)	9.90	(15.85)	(25.64)	(44.55)
Non-trained	359.44	679.42	1165.23	1967.19	3561.84
Share (%)	(4.65)	(8.79)	(15.07)	(25.44)	(46.06)

Note: Values in parentheses are in percentages. The row summation of the shares (percentages) adds up to 100. Exchange rate: US$1 = GHS5.37 (Bank of Ghana, 2019).

### 4.2 Stochastic dominance results

**Fig. 3:**
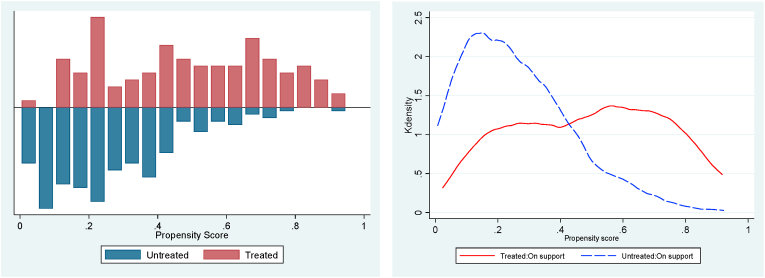
Propensity score density distribution and common support region for adoption.

**Fig. 4:**
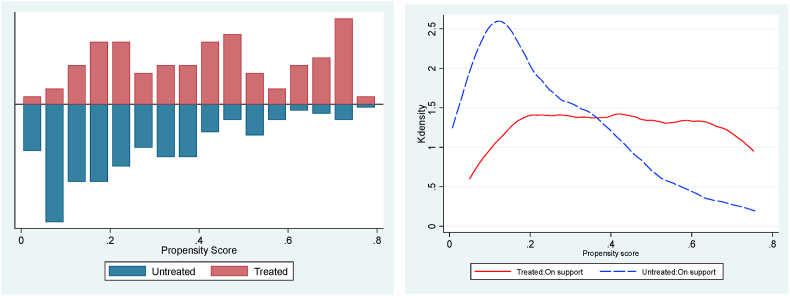
Propensity score density distribution and common support region for cowpea yield.

**Fig. 5:**
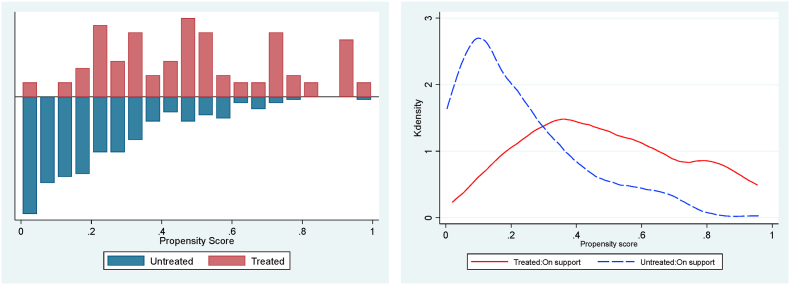
Propensity score density distribution and common support region for cowpea income.

**Fig. 6:**
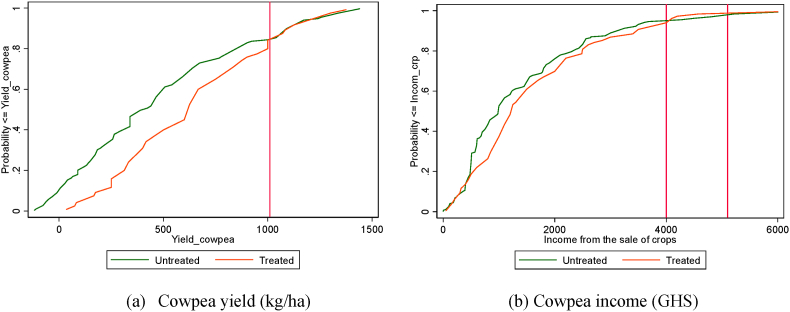
CDF of cowpea yield (a) and cowpea income (b) for trained and non-trained farmers.

The stochastic dominance analysis (SDA) is restricted to households matched based on their propensity score of participation whose distribution and region of common support ranges from 0.0195 to 0.9997 (Fig. 3, Fig. 4, Fig. 5). Households outside the region of common support were dropped. The good overlap between the density distribution of propensity scores for participants and non-participants of the CATP justifies the use of PSM and the basis for comparison using the SDA. Fig. 6 shows the cumulative distribution functions (CDFs) for cowpea yield and crop income for trained and untrained cowpea farmers. The results indicate that yield and crop income of participants stochastically dominate those of non-participants at yields below 1,000 kg/ha of yield and crop income below GHS4,000 (US$745). However, for cowpea yields above 1,000 kg/ha and crop income above GHS5,100 (US$950), the CDFs of both participants and non-participants are almost the same, but the crop income of non-participants stochastically dominate that of the participants for crop income ranging from GHS4,000 (US$745) to GHS5,100 (US$950). The nonparametric Kolmogorov–Smirnov test for first-order stochastic dominance also shows that the CDFs of participants stochastically dominate those of non-participants for cowpea yield and crop income at 1% level of significance. Based on the results, we conclude that conditional on observed characteristics, there is a higher probability that participants in a CATP will, on average, have higher yields and crop income than non-participants.

### 4.3 Impact of training on welfare outcomes: ESR results

Table 5 presents the full information maximum likelihood estimates of the determinants of participation in cowpea training (selection equation) and the impact of participation on adoption of climate-smart cowpea variety, cowpea yield, and crop income (outcome equations). The correlation coefficient (rho_2) in the adoption model is statistically significant while that of the yield and cowpea income model is not statistically significant, suggesting heterogenous results depending on the outcome variable. The exclusion restriction variable, access to a Member of Parliament or politician, is statistically significant in the first stage model (participation in a CATP – Table 5), but insignificant in the outcome equations, thus satisfying the instrument relevance condition. The positive coefficient confirms the expectation that access to a Member of Parliament or politician increases the chance of being included in agricultural development projects. The falsification test established the admissibility of the instrument (Table A1).

**Table 5:** Full information maximum likelihood results of the endogenous switching regression.

Variable	Adoption of climate-smart cowpea			Cowpea yield (kg/ha)			Cowpea income (GHS)		
	Selection	Non-trained	Trained	Selection	Non-trained	Trained	Selection	Non-trained	Trained
Sex	−0.152 (0.191)	0.047 (0.072)	0.062 (0.082)	−0.293 (0.207)	201.581*** (74.667)	160.589* (103.334)	−0.232 (0.210)	0.877*** (0.152)	0.544*** (0.166)
Age	0.036*** (0.011)	−0.012*** (0.004)	−0.013** (0.006)	0.031*** (0.011)	−6.105 (4.959)	2.497 (5.699)	0.033*** (0.011)	−0.027** (0.011)	0.004 (0.010)
Education (years)	0.020 (0.018)	0.005 (0.007)	0.009 (0.007)	0.026 (0.019)	10.889 (7.464)	6.096 (9.182)	0.027 (0.020)	−0.021 (0.016)	0.012 (0.015)
Household size	0.043** (0.019)	−0.001 (0.008)	0.009 (0.009)	0.055*** (0.020)	−5.546 (8.755)	11.858 (10.390)	0.049** (0.020)	0.024 (0.018)	0.004 (0.017)
Farming experience	−0.028*** (0.011)	0.012*** (0.004)	0.007 (0.005)	−0.017 (0.011)	4.322 (4.706)	−9.460* (5.080)	−0.019 (0.011)	0.018* (0.010)	0.012 (0.009)
Cooperative	0.040 (0.258)	−0.024 (0.097)	−0.055 (0.140)	−0.120 (0.281)	39.585 (96.637)	72.701 (171.545)	−0.039 (0.286)	−0.179 (0.200)	0.089 (0.274)
FBO	0.526*** (0.161)	−0.031 (0.064)	−0.091 (0.092)	0.536*** (0.177)	−69.585 (74.965)	−53.603 (101.777)	0.503*** (0.178)	−0.122 (0.156)	0.069 (0.170)
VLSA	0.521*** (0.182)	−0.011 (0.073)	0.063 (0.097)	0.580*** (0.196)	32.225 (84.367)	−197.466* (115.378)	0.531*** (0.202)	0.162 (0.175)	−0.025 (0.180)
Area	0.229* (0.123)	−0.059 (0.052)	0.057 (0.056)				0.363*** (0.132)	0.021 (0.134)	0.193* (0.115)
Tolon district	0.253 (0.344)	−0.683*** (0.135)	−0.186 (0.169)	0.229 (0.357)	809.513*** (124.650)	463.185** (200.674)	0.661* (0.385)	−0.597* (0.310)	−0.815** (0.358)
Savelugu district	1.864*** (0.370)	−0.192 (0.127)	−0.153 (0.226)	1.924*** (0.343)	529.459*** (181.840)	736.831*** (229.182)	1.837*** (0.351)	0.404 (0.411)	0.472 (0.401)
Yendi district	0.279 (0.350)	−0.517*** (0.117)	−0.451*** (0.150)	0.376 (0.331)	35.353 (114.007)	87.541 (188.611)	0.443 (0.331)	−0.255 (0.247)	−0.458 (0.308)
Bawku Municipal	0.795** (0.396)	−0.910*** (0.140)	−0.695*** (0.180)	0.572 (0.374)	1022.657*** (135.844)	882.765*** (205.780)	0.758* (0.391)	−0.414 (0.304)	0.250 (0.341)
Binduri district	0.329 (0.352)	−0.594*** (0.117)	−0.557*** (0.150)	0.333 (0.325)	977.737*** (107.666)	968.355*** (185.651)	0.548 (0.335)	−0.560** (0.247)	−0.195 (0.309)
Wa West district	−0.120 (0.340)	0.081 (0.120)	−0.064 (0.172)	−0.188 (0.350)	53.198 (121.812)	400.819* (217.357)	−0.232 (0.211)	0.067 (0.251)	0.498 (0.345)
Access to Member of Parliament	0.546** (0.216)			0.697*** (0.226)			0.556** (0.231)		
Distance extension	−0.002 (0.011)			−0.007 (0.012)			0.000 (0.012)		
Constant	−3.386*** (0.581)	1.127*** (0.216)	1.299** (0.568)	−3.210*** (0.565)	381.213* (221.573)	208.293 (488.655)	−3.787*** (0.608)	7.248*** (0.545)	5.959*** (1.027)
rho_1		−0.189 (0.539)				0.293 (0.323)			−0.171 (0.454)
rho_2			−0.960*** (0.023)		−0.104 (0.390)			−0.006 (0.465)	
Model diagnostics									
Wald chi2	73.91***			92.30***			103.21***		
Log likelihood	−262.28			−2546.54			−533.96		
LR test $\chi^{2}\left(1\right)$	21.42***			0.77			0.14		
Observations	319			320			320		

***Notes***: Adoption of climate-smart cowpea is a dummy variable (1 = adopters). Sigma is an auxiliary parameter (i.e. the square-root of the variance of the residuals of the endogenous switching regression model). Sigma (participants) associated with yield and income for participants are 0.0637 (0.055) and 410.991 (37.231) and sigma (non-participants) associated with yield and income for non-participants are 0.866 (0.042) and 423.438 (21.774), respectively. Figures in parentheses are standard errors. Exchange rate: US$1 = GHS5.37 (Bank of Ghana, 2019). ***p < 0.01 **p < 0.05 and * p < 0.001.

Results from the selection equation indicate that participation in the CATP is significantly influenced by age, household size, membership in a FBO, membership in a VSLA, farm area, and access to a Member of Parliament or politician. The age coefficient indicates that older household heads are more likely to participate in the CATP relative to younger household heads. Household size has a positive effect on participation in the CATP. Larger household size corresponds with higher food requirements. Thus, participation in the CATP equips the household head to make an informed decision that supports higher productivity and farm income. Membership in a FBO provides an opportunity for farmers to be part of development projects as most development organizations are more likely to work with farmer groups [4]. Several studies [[33], [34], [35]] have highlighted the critical role FBOs play in capacity building and adoption of agricultural technologies. Area under cowpea is associated with a higher probability of participation in the CATP. Farmers with a larger farm are more likely to experiment with new technologies gained from training on a portion of their farmland to verify the economic benefits. In addition, land serves as a proxy for wealth given that land is the main production asset for farmers [36,37]. Farmers with more land are expected to participate in agricultural training activities that provide information on modern inputs to enhance their yield relative to poorer cowpea producing households. Farmers who reside in Tolon, Savelugu, and Bawku Municipal districts are more willing to participate in cowpea training relative to farmers who resides in the Nadowli District. Consistent with the findings and reasons of [36], districts that are more likely to participate in the cowpea training are located closely to national agricultural research systems and non-governmental organizations involved in research, training and advisory services to smallholder farmers.

Table 6 presents the estimated ATT of participation in cowpea training on the adoption of climate-smart cowpea variety, yield, and cowpea income. Compared to the results in Table 3, the ATT accounts for selection bias due to observable and non-observable characteristics between participants and non-participants. The results show that participating in the CATP significantly increases the adoption of climate-smart cowpea varieties and cowpea yield (ATT) by 75% and 119 kg/ha, respectively. The result shows that trained farmers record an estimated adoption rate of 78%, whereas the estimated adoption rate for trained farmers had they not participated (counterfactual adoption) in the training is just 3%. Participants in the CATP report an estimated yield of 888 kg/ha while the estimated yield for participants had they not participated (counterfactual yield) in the cowpea training is 770 kg/ha. The ATT's value of 119 kg/ha represents a yield increase of 15%.^5^ The average cowpea yield of 888 kg/ha by participants under observed conditions is 63% of the average yield (1400 kg/ha) and 36% of the potential yield (2500 kg/ha) obtained from on-farm experiments [38]. For non-participants, the mean cowpea yield would have decreased by 107 kg/ha had they participated in the cowpea training. The expected cowpea income for participants is GHS1,470 (US$274), and the expected income of participants had they not participated (counterfactual income) in the cowpea training is GHS1,182 (US$220). The expected income for non-participants had they participated in the cowpea training is GHS1,350 (US$251) and the expected income for non-participants had they not participated in cowpea training is GHS1,096 (US$204). The ATT indicates that participating in cowpea training increases cowpea income by GHS289 (US$54), which represents an income gain of 24%. The results are consistent with the finding of previous studies by Ref. [18,36,39,40]. Based on the results, it can be concluded that the causal mechanism of higher welfare impacts of training is gained through high levels of adoption of improved cowpea varieties.

**Table 6:** Average expected cowpea income and land productivity by training status.

Sample	Training decision		Treatment effect
	To train	Not to train	
**Adoption (1/0)**			
Trained	0.78	0.03	ATT = 0.75 (0.04)***
Non-trained	0.85	0.62	ATU = 0.23 (0.03)***
Heterogeneity effects	BH_11_ = −0.07	BH_12_ = −0.59	TH_11_ = 0.52
**Yield (kg/hectare)**			
Trained	888.21	769.59	ATT = 118.62 (53.20)**
Non-trained	719.57	826.45	ATU = −106.88 (41.57)**
Heterogeneity effects	BH_21_ = 168.64	BH_22_ = −56.86	TH_21_ = 225.50
**Cowpea income (GHS)**			
Trained	1470.40	1181.84	ATT = 288.56*** (110.85)
Non-trained	1349.61	1096.08	ATU = 253.53***(74.35)
Heterogeneity effects	BH_31_ = 120.79	BH_32_ = 85.76	TH_31_ = 35.03

*Notes*: The numbers in parentheses associated with ATT and ATU are standard errors. Exchange rate: US$1 = GHS5.37 (Bank of Ghana, 2019). ***p < 0.01, and **p < 0.05.

Concerning the adoption of climate-smart cowpea varieties, the results show that base heterogeneity is negative for both participants (BH_11_) and non-participants (BH_12_), while the transitional heterogeneity (TH_11_) is positive. The TH_11_ suggests that the effect of adoption of climate-smart varieties is greater for households that participated in the CATP relative to those that did not participate. Concerning cowpea yield, the effect of the base heterogeneity is positive for participants (BH_21_) and negative for non-participants (BH_22_). BH_21_ suggests that the effect of cowpea training is larger for participants than non-participants had they participated in the cowpea training. BH_22_ suggests that the cowpea yield effect is smaller for participants had they not participated in cowpea training relative to non-participants had they participated in cowpea training. In terms of cowpea income, we observed that the effect of base heterogeneity for participants (BH_31_) is greater than the base heterogeneity effect for non-participants (BH_32_) and is positive for both participants and non-participants. BH_31_ indicates that the effect of cowpea income is larger for participants had they not participated in cowpea training relative to non-participants had they participated in cowpea training. Similarly, BH_32_ indicates that the effect of cowpea income is higher for participants had they not participated in the CATP compared to non-participants had they participated in cowpea training. The transitional heterogeneity effect is positive for both yield and cowpea income. This implies that the effect on cowpea yield and income are larger for households that participated in the cowpea training relative to those that did not participate.

### 4.4 Robustness check

The PSM and IPWRA estimates used as robustness checks for the ESR model are reported in Table 7. The results indicate that adoption of climate-smart cowpea varieties, cowpea yield, and cowpea income are significantly higher for participants compared to non-participants of the CATP. Participation in the CATP increased adoption, cowpea yield, and cowpea income by 17%, 175 kg/ha, and GHS410 (US$76.35), respectively, using the nearest neighbor matching method. Adoption of the climate-smart cowpea variety, cowpea yield, and cowpea income increased by 12%, 176 kg/ha and GHS445 (US$83), respectively, for participants in the CATP when the kernel matching method is employed. The IPWRA estimates also show that participants in the CATP recorded a significantly higher climate-smart cowpea variety adoption, yield, and income than non-participants. Comparatively, the estimates of PSM and IPWRA are consistent with the estimates obtained from the ESR estimation. The results indicate that our findings are robust to different estimation techniques. Finally, we conducted a heterogeneous impact of cowpea training, which is necessary for designing effective and well-targeted training programs for rural farm households. The analysis is reported in appendix B.

**Table 7:** Propensity score matching and IPWRA estimate of cowpea training.

Panel A	Adoption (1/0)		Yield (kg/Hectare)		Cowpea income (GHS)	
	ATT	Robust Std. Error	ATT	Robust Std. Error	ATT	Robust Std. Err.
IPWRA	0.165**	0.082	128.40**	61.44	551.36**	242.15
Panel B	Adoption (1/0)		Yield (kg/Hectare)		Cowpea income (GHS)	
	ATT	Bootstrap Std. Error	ATT	Bootstrap Std. Error	ATT	Bootstrap Std. Error
NNM	0.165**	0.077	174.86**	87.66	410.30**	206.18
KNM	0.121*	0.073	175.92*	97.26	445.22**	213.42

Note: IPWRA is inverse probability weighted regression adjustment; NNM is nearest neighbor matching; and KNM is kernel matching. Exchange rate: US$1 = GHS5.37 (Bank of Ghana, 2019). **p < 0.05 and * p < 0.001.

## 5 Conclusion and policy implications

Agricultural training programs remain one of the mechanisms for introducing and disseminating modern technologies to farm households. Several studies have highlighted the role of training on household welfare based on farmer field schools and farmer-to-farmer extension delivery. In recent years, CATPs are gaining popularity. These training programs require participating farmers to gain informal certification after completing a set of practices ranging from seed selection to postharvest management. However, there is limited evidence on the socio-economic impact of these intensive agricultural training programs. This paper makes an empirical contribution in addressing the paucity of information regarding the impact of intensive agricultural training capacity building in northern Ghana. Using farm household level data from the region and an ESR model, this study evaluates the impact of participation in cowpea training on the adoption of climate-smart cowpea varieties, yield and cowpea income.

The results show that participation in the CATP is significantly influenced positively by age, household size, membership in a FBO, membership in a VSLA, farm area, and access to Member of Parliament or a politician. However, farming experience is negatively associated with participation in cowpea training. The result implies that future CATPs in northern Ghana must consider these factors in the selection process to ensure higher participation.

Results from the ESR model indicate that after accounting for both observed and unobserved heterogeneity, participation in the CATP is associated with a positive effect on the adoption of climate-smart cowpea varieties, yield, and cowpea income. Gains due to participation in cowpea training on good agronomic practices are 75% for the adoption of improved cowpea varieties, 118.26 kg/ha for cowpea yield, and GHS288.56 (49.88 USD) for cowpea income.

The positive effect of cowpea training on welfare outcomes implies that continuous support and scaling-up of agricultural training programs in good agronomic practices and commercial orientation will enhance greater participation and impact on the welfare of farm households in Ghana. Agricultural development programs that enhance participation in CATPs must be promoted and sustained beyond the duration of commissioned projects or programs. Creating awareness and eliminating barriers to participation in CATPs can induce greater participation and enhance the adoption of improved varieties and welfare effects among farmers in northern Ghana.

## Figures and Tables

**Table A1:** Test of the validity of the instrument (falsification test)

Variable	Cowpea training (dummy)		Adoption of improved varieties (dummy)		Cowpea yield (kg/ha)		Cowpea income (GHS)	
	Coefficient	Robust	Coefficient	Robust	Coefficient	Robust	Coefficient	Robust
		Std. Error		Std. Error		Std. Error		Std. Error
Access to MP	0.559**	0.240	0.167	0.168	31.144	65.300	−64.531	173.670
Constant	−3.777***	0.552	0.631*	0.149	838.234***	165.703	689.953	365.460
	LR chi2(1) = 5.43**		F value = 0.99		F value = 0.23		F value = 0.14	

Notes: MP refer to Member of Parliament. Exchange rate: US$1 = GHS5.37 (Bank of Ghana, 2019). ***p < 0.01 and **p < 0.05.

**Fig. B1:**
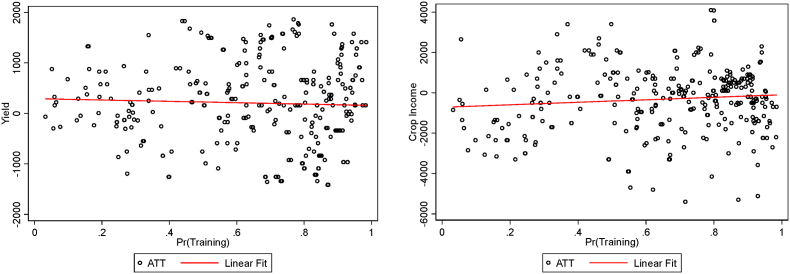
Heterogeneity of treatment effects over propensity scores.

**Table B1:** Heterogeneous treatment effects of cowpea training

Variables	Adoption	Yield	Cowpea income
Sex	0.036 (0.139)	73.893 (155.538)	976.246** (445.241)
Age	−0.019*** (0.006)	5.385 (8.448)	−20.989 (16.764)
Education (years)	0.004 (0.013)	1.846 (14.384)	−42.047 (37.943)
Household size	0.006 (0.011)	9.764 (14.759)	−69.925** (34.837)
Farming experience	0.009 (0.006)	−10.256 (6.892)	25.093* (13.970)
Cooperative	−0.230 (0.210)	14.330 (291.200)	860.122 (844.646)
FBO membership	−0.121 (0.123)	−44.601 (156.540)	−639.602* (333.970)
VLSA membership	−0.012 (0.134)	−289.467* (164.954)	−906.748** (417.323)
Cowpea area	0.063 (0.090)	−149.139 (89.752)	90.824 (245.969)
Tolon district	−0.507 (0.327)	152.321 (366.088)	−389.858 (873.060)
Savelugu district	−0.645*** (0.228)	991.714*** (229.534)	−90.289 (826.117)
Yendi district	−0.813*** (0.230)	100.919 (278.174)	269.482 (895.259)
Bawku Municipal	−1.006*** (0.257)	889.147** (340.825)	810.725 (882.979)
Binduri district	−1.172*** (0.255)	1119.241*** (281.870)	−85.629 (972.452)
Wa West district	−0.140 (0.283)	634.377** (272.945)	977.651 (886.890)
Access to Member of Parliament	−0.038 (0.160)	444.682** (173.193)	−245.270 (502.179)
Distance to extension	0.007 (0.005)	−1.948 (8.293)	9.020 (17.911)
Constant	1.287*** (0.389)	−302.315 (518.898)	1128.166 (1089.606)
Observations	109	109	109
R-squared	0.360	0.372	0.349

Note: The dependent variable is the ATT of each respective outcome indicators. Robust standard errors are in parentheses. Exchange rate: US$1 = GHS5.37 (Bank of Ghana, 2019). ***p < 0.01, **p < 0.05, and * p < 0.1.

## Appendix B Heterogeneous treatment effect of cowpea training

### Heterogeneous treatment effects

#### Heterogeneous treatment effect over propensity scores

Fig. B1 shows the variation of ATT for yield and income scores over the estimated propensity scores. The results show that the ATT for cowpea yield and income varies significantly with the propensity. Moreover, the slope is negative for yield and positive for income. The positive slope suggests that the effect of the CATP on crop income is stronger for households with the highest propensity to participate in cowpea training. On the other hand, the negative slope suggests that the effect of training on cowpea yield is lower for households with the highest propensity to participate in cowpea training.

#### Heterogeneous effects of cowpea training over household characteristics

Despite the positive impact of participation in the CATP on the welfare outcomes of rural households, we argue that welfare indicators may differ among different farm households. Understanding the heterogeneous impact of cowpea training is necessary for designing effective and well-targeted training programs for rural farm households. Following the approach by Refs. [33], we used the ATT of individual outcome variables (adoption, yield and income) as a dependent variable in an OLS regression and ascertain how socio-economic characteristics explain variations in the estimated ATT. The results are reported in Table B1.

In terms of the adoption of climate-smart cowpea varieties, we find statistically significant differential effect of the CATP among treated households with respect to age and residence in Savelugu, Yendi, Bawku Municipal, and Binduri districts. The results indicate that cowpea training exerts a lower effect for older farmers and farmers located in these districts. The estimated OLS results show that to a large extent, the CATP has heterogeneous effects given that membership in a VSLA, district dummies, and access to a Member of Parliament or a politician significantly influence the estimated ATT for cowpea yield among the treated farm households. The results indicate that being a member of a VSLA reduces the probability of benefiting from cowpea training. However, access to political figures and households who reside in Savelugu, Yendi, Bawku Municipal, Binduri, and Wa West districts benefit most from cowpea training. This suggests that increasing farmers’ access to agricultural training can maximize cowpea yield. With respect to cowpea income, we find a statistically significant differential effect of cowpea training among treated households with respect to sex, household size, farming experience, member of a VSLA and member of a FBO. The results show that male-headed households and farmers who have several years of experience in cowpea farming benefit most from the CATP. This implies that identifying experienced cowpea farmers and using them as model farmers in technology dissemination may result in higher impacts on income.

## Footnotes

1 The sampling frame consists of all cowpea producing districts in (former) northern Ghana consisting of Northern, Upper East, and Upper West regions. The zone is made up of 52 districts with 26 in Northern, 15 in Upper East and 11 in Upper West regions. For more details on the top 10 cowpea producing districts, refer to the Ministry of Food and Agriculture (MoFA). (2017). Agriculture in Ghana Facts and Figures, 2016. Statistics, Research and Information Directorate (SRID) of MoFA, Accra. https://new-ndpc-static1.s3.amazonaws.com/CACHES/PUBLICATIONS/2016/04/16/AGRICULTURE-IN-GHANA-Facts+and+Figures-2010.pdf.

2 The power of the test is the chance to reject the null hypothesis, given that the null hypothesis is false (i.e. given that the alternative hypothesis is true). It is the used to determine the sample size for a study.

3 The exchange rate at the time of the survey in July 2019 is US$1 = GHS5.37 (Bank of Ghana, 2019).

4 A falsification test certifies the admissibility of the selection instrument as a valid instrument: If a variable is an appropriate selection instrument, it will affect the choice of participation in the CATP, but it will not affect the welfare or outcomes variables [28]. Our results indicate that the instrument can be considered as valid given that it is statistically significant in the participation in the CATP equation (equation (2)) but not significant in the outcome equations (equation (4b)) (Table A1).

5 The percentage yield is computed as the ratio of the ATT (119 kg/ha) to the yield under the counterfactual scenario (770 kg/ha) and this is expressed as a percentage.
