# Supplementary material for: Climate-smart cowpea adoption and welfare effects of comprehensive agricultural training programs
Source: Technol Soc. 2021 Feb;64:101468. doi: 10.1016/j.techsoc.2020.101468 (PMC7973722; doi:10.1016/j.techsoc.2020.101468)
Supplement: Multimedia component 1 [file mmc1.docx]

**Climate-smart cowpea adoption and welfare effects of comprehensive agricultural training programs**

**Abstract**

Agricultural training programs remain one of the primary mechanisms for disseminating modern and climate-smart technologies with the aim to improve the welfare outcomes of smallholder farmers. With persistent low agricultural productivity in Sub Saharan Africa (SSA), the content, effectiveness, and mode of delivery of training programs remain a debate. In this paper, we examine the adoption, productivity, and income effects of participating in a novel comprehensive agricultural training program (CATP) involving cowpea farmers in northern Ghana by using the endogenous switching regression (ESR) model. The CATP requires farmers to complete a set of modules on good agronomic practices to gain informal certification. The results indicate that participating in the CATP increases the adoption of climate-smart cowpea varieties, productivity, and income by 75, 15, and 24 percentage points, respectively. These positive welfare effects of participating in the CATP confirm the need to increase capacity-enhancing activities in agricultural development projects, and design mechanisms to eliminate barriers to participation among poor rural farm households.

**Keywords**: Comprehensive training programs; climate-smart cowpea; endogenous switching regression; adoption; welfare effects.

**1. Introduction**

In developing countries, most households depend on the agricultural sector for livelihood support. Yet, persistent low agricultural productivity and limited transformation of the food system remain significant hurdles to improving welfare [1-2]. Several past and current development interventions prioritize increasing agricultural productivity as a viable pathway to improving farmers’ welfare. These interventions include increasing access to technologies such as fertilizer, improved seed varieties, and technical knowledge [3-4]. Agricultural training programs that boost human capital remain one of the mechanisms for improving the knowledge of farmers on emerging technological innovations [5-6]. However, there is limited consensus on the effectiveness of existing training approaches. For example, whereas the training and visit agricultural extension approach is often criticized as a top-down approach, there is limited agreement on the effectiveness of other models, such as farmer field schools (FFS) [7]. While some empirical analysis has found positive impacts of FFS on yield and income, other studies suggest the opposite [7-9]. This study aims at contributing to evidence-based research on the impact of a comprehensive agricultural training programs (CATP) on the adoption of climate-smart cowpea varieties, yield, and income.

Improving the technical skills of farmers using appropriate training methods is particularly crucial in Sub Saharan Africa (SSA) where agricultural productivity and farm incomes are low (). Despite the growing body of evidence, the number of capacity building interventions in Africa outweighs the number of studies that assess the effects of such interventions on the welfare of recipients [8]. A good proportion of studies have focused on FFS, farmer-to-farmer extension, or the use of model farmers [10, 8]. In this study, we argue that despite the importance of these approaches, farmer participation may not necessarily translate into capacity building. Farmers’ participation in agricultural programs may be active or passive depending on several factors, such as education level, income, and social networks [4, 11]. Moreover, it is possible that farmers do not complete all of the training module (e.g., modules from seed selection to postharvest). In such circumstances, analyzing the impact of training on welfare may not be an accurate reflection given that full and partial participants may have different outcomes when compared to non-participants.

A multidisciplinary research team from the Savanna Agricultural Research Institute (SARI) of Ghana’s Council for Scientific and Industrial Research (CSIR) initiated a community training school in 2017. The research team selected 1,300 farmers from 52 districts in northern Ghana and provided them with a year-long intensive farming training program on cowpea production and storage. The specific training modules included identification of quality seed, storage of cowpea seed, use of inoculant, planting (timing and method of planting, namely, row planting and spacing), effective weed management practices, pest management practices, soil fertility management methods, fertilizer application (method, quantity, and timing), suitable harvesting methods (timing and methods), and postharvest management practices (proper storage methods, such as using hermetic or triple-layered bags). The research team in collaboration with local extension officers, combined both practical field demonstration and formal adult learning techniques to train selected farmers. Local extension officers act as a backstop and are a sustainable source of agricultural information. Strict protocols were discussed and adopted by the research team and participating farmers to ensure compliance and active participation. In addition, routine monitoring of the training program was carried out by local extension officers.

Some earlier studies on the impacts of training programs did not explicitly address potential biases associated with non-random program placement and the selection of farmers [9]. Such studies do not adequately control for observed and unobserved differences that exist between trained and untrained farmers, thereby making it difficult to draw conclusive statements given that communities, for example, may be chosen to participate in training programs because of fertile lands or conduciveness for crop production. Similarly, farmers who opt to participate in training programs may be those who are naturally more productive. Hence, a comparison of participants against non-participants may not provide a fair basis for constructing counterfactuals. A recent study by [10] using panel data showed that farmer-to-farmer training increased adoption of improved rice and yields among early trainees while subsequently trained farmers caught up belatedly. This study complements and expands existing empirical analyses by exploring how comprehensive agricultural training provided by a research institution impacts climate-smart cowpea adoption, productivity, and income. Currently, the authors are not aware of any empirical study in Ghana that evaluates the impact of a novel training program on the welfare of rural households.

This analysis evaluates the impact of a CATP on the adoption of climate-smart cowpea varieties, yield, and cowpea income. In the absence of baseline information, the paper uses the endogenous switching regression (ESR) model to deal with both selection bias and unobservable factors that can lead to biased results. Subsequently, we estimate propensity score matching and inverse probability weighted regression adjustment as robustness checks. Our empirical estimates show that completing cowpea training modules increases adoption of climate-smart cowpea varieties, cowpea yield, and income by 75, 15, and 24 percentage points, respectively.

The rest of the paper proceeds as follows. In the next section, we provide a description of our data generation process and the variables in the model. Section 3 consists of our conceptual framework and estimation strategies, as well as a description of our main method of analysis, the endogenous switching regression model. We present and discuss our empirical estimates in Section 4 before presenting the conclusions and policy implications of our study in Section 5.

**2. Data**

This study uses farm household survey data collected in 2019 for the 2018/2019 cropping season from 320 cowpea farmers in seven major cowpea growing districts (Tolon, Savelugu, Yendi, Bawku Municipal, Binduri, Wa West, and Nadowli) in northern Ghana. This study forms part of a more extensive study commissioned by ICRISAT and IITA under the project “Accelerated Varietal Improvement and Seed Delivery of Legumes and Cereals in Africa (AVISA).” The AVISA project aims at refocusing its work to improve the Consortium of International Agricultural Research Centers (CGIAR) and crucial National Agricultural Research Systems (NARS) breeding and seed delivery systems. The AVISA initiative targets the most critical dryland cereals (sorghum and pearl millet) and legume crops (groundnut, common bean, and cowpea) on the African continent as part of the Bill and Melinda Gates Foundation (BMGF) focus geographies where these crops are important (Burkina Faso, Ghana, Mali, Nigeria, Tanzania, and Uganda). Capacity building is a significant component of the AVISA intervention. Fig. 1 presents the map of the study area showing the location (districts) of the farmers interviewed.


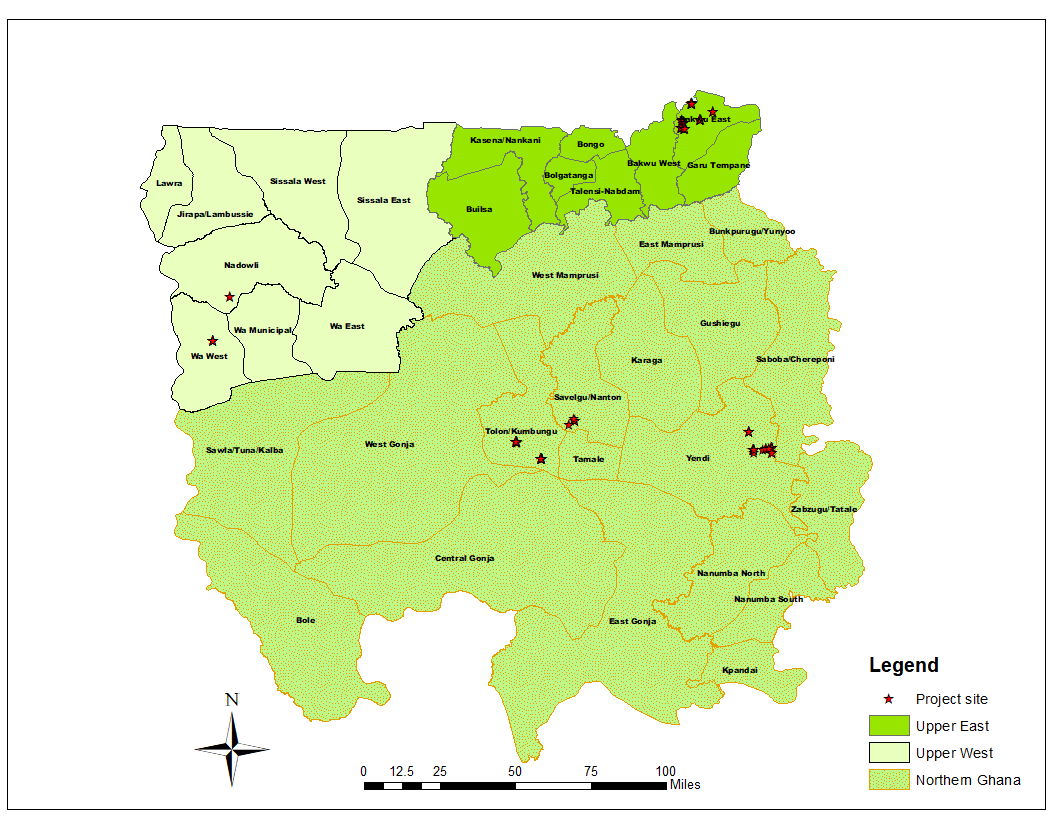


Fig. 1. Study area indicating the data collection sites in northern Ghana

The sampling^[[1]](#footnote-1)^ procedure followed a multi-stage sampling technique to select 320 farmers in the Guinea and Sudan Savannah agro-ecological zones consisting of the Northern, Upper East, and Upper West regions of northern Ghana. In the first stage, seven districts were purposively selected from these three regions based on the quantity of cowpea produced, as well as the accessibility and presence of active Farmer-based Organizations (FBOs). In the second stage, 20 communities were purposively selected from the Northern Region and 10 communities each from the Upper East and Upper West regions from a list of cowpea-producing communities in each of the selected districts based on the volume of cowpea production. Within the selected communities, eight cowpea producers were randomly selected from a list of cowpea producers. In all, 320 cowpea producers were selected from 40 communities within seven districts (Table 1).

A power test^[[2]](#footnote-2)^ was conducted on the study’s sample. Given the intended power, we can derive the required sample size; and given the intended sample size, we can derive the resulting power. Following [12]: *n* = (*N*/1+*N*e^2^), where *N* denotes the total population of farmers in northern Ghana, i.e., 4,228,116 farmers consisting of 2,479,461 from the Northern Region, 1,046,545 from the Upper East Region, and 702,110 from the Upper West Region [13]; *e* denotes the margin of error of the sample, namely, 0.05 (95% confidence level), and *n* denotes the sample size. The derived margin of error of the sample is 6% when the sample is 320 (implying a 94% confidence level). The result implies that we are 94% confident in the results obtained from the sample used for this study. Therefore, the sample is representative and the results can be generalized for the population of farmers in the major cowpea-producing areas of northern Ghana.

Table 1. Distribution of sampled cowpea farmers by region

| Region | # of Districts | # of Communities | # of Households |
| --- | --- | --- | --- |
| Northern | 3 | 20 | 160 |
| Upper West | 2 | 10 | 80 |
| Upper East | 2 | 10 | 80 |
| Total | 7 | 40 | 320 |

The process of data collection commenced with pre-testing of the survey instrument with feedback from the field interview used to refine the survey instrument. Enumerators were recruited, trained, and deployed to communities to conduct a household survey. The data used in this study contain detailed household demographic and socio-economic characteristics, such as sex, education, age, marital status, household size, nativity, and residential status. Farm characteristics include farming experience, farm size, type of crops cultivated, area owned and area under cultivation (including male and female-owned fields), and common fields. Social capital-related variable were captured, namely, membership in a FBO, village savings and loan association (VSLA), or cooperative, as well as access to a Member of Parliament or political figure. Data on access to institutional and social amenities, such as access to agricultural extension services, district assembly, district capital, and market (factor and output) were also collected. Lastly, the survey captured data on participation in cowpea training.

The mean and standard deviations of the variables used in the regression analysis and their definitions are presented in Table 2. Our choice of variables is based on the existing literature [3,4,7,10,11, 14, and 15]. The study captures the welfare of the household in terms of yield, measured in kilograms per hectare, and cowpea income, expressed in Ghana cedi (GHS). Participation in cowpea training is expected to build human capital that will translate into adoption of climate-smart cowpea varieties, higher yields, and therefore an increase in household crop income. The average adoption, yield, and cowpea income are 67%, 847.46 kg/ha, and GHS1486.31 (US$277), respectively. About 34% of sampled households participated in the cowpea training. The average farm household head is relatively young (44 years), belonging to the economically active age group and can therefore be expected to work for the next two decades. On average, 59% of farm households are headed by males. Household heads have an average of seven years of formal education and 20 years of farming experience. Household size is relatively large with an average of nine household members. With respect to social capital, about 19%, 53%, and 46% of farmers are members of a cooperative, FBO, and VSLA, respectively. The area under cowpea cultivation for the sampled farm household is 1.18 hectares and farmers travel an average distance of 3.85 kilometres to access extension information. About 25% of farm households have access to a Member of Parliament or political figure, which proxies for access to both agricultural and other social safety programs promoted by the Government of Ghana with financial support from development partners.

Table 2: Variable name, description, and summary statistics

| Variable | Definition | Mean | SD |
| --- | --- | --- | --- |
| *Dependent variable* |  |  |  |
| Adoption | Adoption of improved cowpea varieties (1 = adoption) | 0.67 | 0.47 |
| Yield | Cowpea yield (kg/ha) | 2,118.65 | 1,488.93 |
| Crop Income | Cowpea income (GHS) | 1,486.31 | 1,227.99 |
| *Treatment variable* |  |  |  |
| Training | Participated in cowpea training (1 = yes) | 0.34 | 0.47 |
| *Explanatory variables* |  |  |  |
| Age | Age of household head (number of years) | 43.68 | 12.40 |
| Sex | Sex of household head (1=male) | 0.59 | 0.49 |
| Education | Number of years of formal education | 6.98 | 4.68 |
| Size | Household size | 9.38 | 4.51 |
| Experience | Years of farming experience | 20.19 | 11.80 |
| Cooperative | Member of cooperative (1 = yes) | 0.19 | 0.39 |
| FBO | Member of a farmer-based organization (1 = yes) | 0.53 | 0.50 |
| VSLA | Member of a village savings and loan association (1 = yes) | 0.46 | 0.50 |
| Cowpea area | Area under cowpea (hectares) | 1.18 | 0.76 |
| Parliament member | Access to a Member of Parliament or political figure (1 = yes) | 0.25 | 0.43 |
| Extension | Distance to nearest extension service (km) | 3.85 | 7.85 |
| *District level controls* |  |  |  |
| Tolon district | Household is located in Tolon district (1 = yes) | 0.15 | 0.36 |
| Savelugu district | Household is located in Savelugu district (1 = yes) | 0.18 | 0.38 |
| Yendi district | Household is located in Yendi district (1 = yes) | 0.18 | 0.38 |
| Bawku Municipal | Household is located in Bawku district (1 = yes) | 0.09 | 0.29 |
| Binduri district | Household is located in Binduri district (1 = yes) | 0.16 | 0.37 |
| Wa West | Household is located in Wa West district (1 = yes) | 0.13 | 0.33 |
| Nadowli district | Household is located in Nadowli district (1 = yes) | 0.13 | 0.33 |

Note: Exchange rate: 1 US$=GHS5.37 (Bank of Ghana, 2019).

**3. Methodology**

*3.1 Conceptual framework and estimation strategies*

Participation in a CATP and its effect on the adoption of improved varieties, yield, and income are considered to be a stepwise decision-making process. The decision to participate in a CATP is a behavioral response and thus modelled as a random utility function, where a set of alternatives and constraints are faced by the decision-maker [14-15]. A risk-neutral utility maximizing cowpea grower, $i$ decides to participate in a CATP if the utility derived from training ($U_{iA}$) exceeds that from otherwise ($U_{iN}$), such that the difference ($D_{i}^{*}$) between the two states is given by:

$D_{i}^{*}=U_{iA}-U_{iN}>0. (1)$

$D_{i}^{*}$ is a latent variable that captures the expected benefit from the choice of participation in a CATP concerning non-participation and can therefore be expressed as a function of observable components in the latent variable model below:

$D_{i}^{*}=Z_{i}\alpha+\varepsilon_{i}>0 where D_{i}=\left\{ \begin{aligned} 1 if D_{i}^{*}>0 \\ 0 otherwise \end{aligned} \right. (2)$

where $D$ is a binary decision variable that equals one if a cowpea farmer chooses to participate in a CATP and equals zero otherwise; $Z$ is a vector of household demographics, socio-economic, and farm-level characteristics; $\alpha$ is a vector of parameters to be estimated; and $\varepsilon_{i}$ is a random error term. In this paper, we define a cowpea trained farmer as any cowpea farmer who has completed all modules of the CATP.

Participation in a CATP is expected to influence the adoption of improved varieties, yield, and cowpea income. The outcome variable is expressed as a function of a vector, $X$, of variables and an endogenous adoption variable ($D$), such that:

$$Y_{l}=\omega X_{i}+\delta D_{i}+\mu_{i,} (3)$$

where $Y_{i}$ represents the outcome variables (adoption of improved cowpea varieties, yield, and cowpea income), $D$ is the participation variable defined previously, $\omega$ and $\delta$ are parameters to be estimated, and $\mu$ is an error term. Since farmers were not randomly assigned, estimates of $\delta$ will be biased. Trained farmers may be systematically different from non-trained farmers, which may bias the actual effect of training on the outcome variables [16]. Estimating equation (3) with ordinary least squares (OLS) produces biased estimates. Due to this shortcoming, several methods have been proposed for non-experimental data including propensity score matching (PSM), inverse probability weighted regression adjustment (IPWRA), instrumental variables, and endogenous switching regression (ESR) models. To correct this biased estimate, we employ an ESR model to address the endogeneity in participation in cowpea training due to self-selection. Nonetheless, we use both the PSM and IPWRA as robustness checks. Details of the empirical frameworks for PSM and IPWRA can be found in [3, 17], respectively.

*3.2 Endogenous switching regression (ESR)*

Several studies have employed the ESR model [18-22]. Following [18], the ESR model consists of separate outcome equations for trained and non-trained farmers, conditional on the choice of cowpea training decisions, namely:

Regime 1 (Trained in CAT): $Y_{1i}=\beta_{1}X_{1i}+e_{1i} if D_{i}=1 and (4a)$

Regime 2 (Untrained in CAT): $Y_{2i}=\beta_{2}X_{2i}+e_{2i} if D_{i}=0, (4b)$

where $Y_{1i}$ and $Y_{2i}$ are the outcome variables for trained and non-trained, $X_{1i}$ and $X_{2i}$ are household and farm-level characteristics; $\beta_{1}$ and $\beta_{2}$ are parameters to be estimated for trained and non-trained farmers regimes, respectively; and $e_{1}$ and $e_{2}$ are random disturbance terms.

For the ESR model to be identified, the variables in the choice model (equation 2) must contain an exclusion restriction variable [23]. Following the example of [24-26], we use access to a member of parliament or whether a political figure resides in the constituency, district, or community as an instrument. Members of Parliament or political figures can dictate the placement of developmental projects in their areas of jurisdiction and farmers who are related to or have access to such political figures are more likely to be included in agricultural development projects. This variable is likely to be correlated with the choice of participation in a CATP, but is unlikely to have any direct effect on the outcome variables. The admissibility of the instrument is established by performing a simple falsification test^[[3]](#footnote-3)^ following the approach of [22].

Following the specification of [3], the error terms ($\varepsilon_{i}$, $e_{1i}$, and $e_{2i}$) are assumed to have a trivariate normal distribution with mean vector zero and covariance matrix:

$$cov\left( \varepsilon, e_{1}, e_{2} \right)=\binom{\begin{matrix} \begin{matrix} \sigma_{\varepsilon}^{2} & \sigma_{e_{1}\varepsilon} & \sigma_{e_{2}\varepsilon} \end{matrix} \\ \begin{matrix} \sigma_{e_{1}\varepsilon} & \sigma_{e_{1}}^{2} & \sigma_{e_{1}e_{2}} \end{matrix} \end{matrix}}{\begin{matrix} \sigma_{e_{2}\varepsilon} & \sigma_{e_{1}e_{2}} & \sigma_{e_{2}}^{2} \end{matrix}}, (5)$$

where $\sigma_{\varepsilon}^{2}$ = var ($\varepsilon$), which is assumed to be 1 since $\gamma$ is only estimable up to a scale factor [27], $\sigma_{e_{1}}^{2}$ = var ($e_{1}$), $\sigma_{e_{2}}^{2}$ = var($e_{2}$), $\sigma_{e_{1}\varepsilon}$ = cov ($e_{1},\varepsilon$), and $\sigma_{e_{2}\varepsilon}$ = cov ($e_{2},\varepsilon$). The covariance between $e_{1}$ and $e_{2}$ is not defined since $Y_{1}$ and $Y_{2}$ are never observed simultaneously [27]. The expected values of the error terms $e_{1}$ and $e_{2}$ can be expressed as:

$$E\left( e_{1} | D_{i}=1 \right)=\sigma_{e_{1}\varepsilon}\lambda_{1} and (6)$$

$$E\left( e_{2} | D_{i}=0 \right)=\sigma_{e_{2}\varepsilon}\lambda_{2} , (7)$$

where $\lambda_{1}$ and $\lambda_{2}$ are the inverse mils ratio (IMR) calculated from the selection equations (2) and included in the outcome equations (4a) and (4b) to correct for selection bias in a two-step estimation procedure known as the endogenous switching treatment regression model [27]. The new outcome equations for the two regimes can be specified as:

$$Y_{1i}=\beta_{1}X_{1i}+\sigma_{e_{1}\varepsilon}\lambda_{1}+\omega_{1} if D_{i}=1 and (8)$$

$$Y_{2i}=\beta_{2}X_{2i}+\sigma_{e_{2}\varepsilon}\lambda_{0}+\omega_{2} if D_{i}=0. (9)$$

If $\sigma_{e_{1}\varepsilon}$ and $\sigma_{e_{2}\varepsilon}$ in equations (8) and (9) are statistically significant, then there is endogenous switching; otherwise, there is exogenous switching. The full information maximum likelihood (FIML) estimation approach is used by the study to estimate the selection and outcome equations simultaneously. This method is more efficient than the two-step procedure [29-30]. The coefficients from the ESR model can be used to derive the average treatment effect on the treated (ATT) and the average treatment effect on the untreated (ATU) by comparing the expected values of the outcomes of participation and non-participation in actual and counterfactual scenarios. The ATT, ATU, base heterogeneity (BH), and transitional heterogeneity (TH) are calculated following [22] and [23]. Detailed representations of the calculations are reported in [3].

*3.3 Non-parametric regression and Stochastic dominance analysis*

The study employs a non-parametric local polynomial regression to establish the existing relationship between the outcome variables and the CATP. We also use the stochastic dominance analysis (SDA) to compare the cumulative distributions of adoption, cowpea yields, and income among participants and non-participants of the CATP. This method focuses on the distribution of the mean and the variance of the economic measures (adoption, yield, and income) for households matched based on their propensity score of participation within the region of common support. This paper used the first two orders of stochastic dominance to differentiate between participation and non-participation in cowpea training. Following the definitions by [31], the first-degree stochastic dominance (FSD) order states that if one cumulative distribution is to the left of another cumulative distribution for all levels of the outcome variable, then participating households with the distribution to the right would dominate those to the left. The second stochastic dominance (SSD) order assumes that human beings are risk averse and prefer to avoid lower outcomes. Graphically, a technology would be dominant in the analysis if the area under its cumulative probability curve is smaller at every outcome level compared to the curve of the alternative [31]. The test for first-order stochastic dominance is conducted using the nonparametric Kolmogorov–Smirnov test.

**4. Results and Discussion**

*4.1 Descriptive statistics*

Table 3 reports the mean values of our outcome and explanatory variables by training status. The results indicate that trained farmers recorded significantly higher adoption (78% vs. 62%) of climate-smart cowpea varieties and obtained higher yields (888.13 Kg/ha vs. 826.45 kg/ha) compared to non-trained farmers, although the difference in yields is not statistically significant. Trained farmers recorded statistically significantly higher cowpea income (US$307) than non-trained farmers (US$261). Concerning the explanatory variables, trained farmers were significantly distinguishable in terms of age, member of a FBO, member of a VSLA, area under cowpea cultivation, and location (Savelugu and Wa West districts). Trained household heads are on average four years older than non-trained household heads. About 15% and 11% more trained household heads belong to a FBO and VSLA, respectively, compared to non-trained household heads. The area under cowpea cultivation controlled by trained farmers is 0.41 hectares more than non-trained farmers.

Table 3: Descriptive statistics by training status

| Variable | Full Sample | Trained (T) | Non-trained (N) | Difference (T-N) |
| --- | --- | --- | --- | --- |
| *Dependent variable* |  |  |  |  |
| Climate-smart variety (%) | 0.67 | 0.78 | 0.62 | 0.16*** |
| Yield (kg/ha) | 847.46 | 888.13 | 826.45 | 61.68 |
| Crop Income (GHS) | 1,486.31 | 1,648.30 | 1,402.63 | 245.67* |
| *Explanatory variables* |  |  |  |  |
| Age | 43.68 | 46.58 | 42.18 | 4.40*** |
| Sex | 0.59 | 0.58 | 0.60 | -0.02 |
| Education | 6.98 | 7.06 | 6.93 | 0.13 |
| Size | 9.38 | 9.88 | 9.12 | 0.76 |
| Experience | 20.19 | 21.63 | 19.45 | 2.19 |
| Cooperative | 0.19 | 0.15 | 0.21 | -0.06 |
| FBO | 0.53 | 0.63 | 0.48 | 0.15*** |
| VSLA | 0.46 | 0.53 | 0.42 | 0.11* |
| Cowpea area | 1.18 | 1.45 | 1.04 | 0.41*** |
| Parliament member | 0.25 | 0.28 | 0.23 | 0.06 |
| Extension | 3.85 | 4.49 | 3.53 | 0.96 |
| Tolon district | 0.15 | 0.13 | 0.16 | -0.03 |
| Savelugu district | 0.18 | 0.33 | 0.09 | 0.25*** |
| Yendi district | 0.18 | 0.17 | 0.18 | -0.01 |
| Bawku Municipal | 0.09 | 0.08 | 0.09 | -0.01 |
| Binduri district | 0.16 | 0.12 | 0.18 | -0.06 |
| Wa West | 0.13 | 0.07 | 0.15 | -0.08** |
| Nadowli district | 0.13 | 0.09 | 0.14 | 0.05 |

Note: Exchange rate: 1 US$=GHS5.37 (Bank of Ghana, 2019).

Fig. 2 shows the result of the non-parametric local polynomial regression that establishes a relationship between the following: adoption of climate-smart cowpea variety and area for trained farmers (panel A), adoption and area for non-trained farmers (panel B); cowpea yield and area for trained farmers (panel C), cowpea yield and area for non-trained farmers (panel D); cowpea income and area for trained farmers (panel E); and cowpea income and area for non-trained farmers (panel F). A smooth, positive trend is observed between adoption and area cultivated among trained farmers while a fluctuation is observed among non-trained farmers. The result highlights the importance of training in technology adoption. Consistent with the literature [3, 32], we observed a negative relationship between cowpea yield and area under cultivation for trained and untrained farmers who cultivate less than two hectares. This result indicates that farm households with relatively small landholdings are more likely to increase their productivity, especially in areas where mechanization support is absent. Panel E shows that among trained farmers, cowpea income and area exhibit a positive relationship for all areas of cowpea, but the observed pattern is different for non-trained cowpea farmers (panel F). The positive relationship only holds for non-trained farmers that have below 2.8 hectares. Cowpea income decreases with farm size for non-trained farmers who own more than 2.8 hectares under cowpea cultivation. No conclusive statement can be inferred given that other factors that might influence adoption, yield, and income are not controlled in the local polynomial regression.

Panel A: Adoption vs. area for trained farmers Panel B: Adoption vs. area for non-trained Panel

Panel C: Yield vs. area for trained farmers Panel D: Yield vs. area for non-trained farmers

Panel E: Income vs. area for trained farmers Panel F: Income vs. area for non-trained

Fig. 2: Local polynomial regressions for trained and non-trained cowpea farmers.

Table 4 shows the quintile distribution of adoption, cowpea yield, and income disaggregated by participation in the CATP. Trained farmers recorded a consistently higher rate of adoption of climate-smart cowpea varieties than non-trained farmers, with the majority of the trained farmers within the third and fourth quintiles of the adoption distribution. Apart from the second and fourth quintiles of yield distribution, trained farmers recorded higher yields than non-trained farmers. However, the fifth quintile of non-trained farm households did not record any yield indicating a share of zero. Similarly, trained farmers have a relatively higher income than non-trained farmers. The first quintile (poorest) of the sample farm households recorded 4% (trained) and 5% (non-trained) of the total cowpea income relative to the fifth quintile (richest) who recorded 46% of the total cowpea income. The poorest trained farmers recorded higher yields than the poorest non-trained farmers, but the poorest non-trained farmers received relatively higher cowpea income than the poorest trained farm households.

Table 4: Distributional summary statistics for adoption, yield, and cowpea income

| Quintiles | First | Second | Third | Fourth | Fifth |
| --- | --- | --- | --- | --- | --- |
|  | Quintile | Quintile | Quintile | Quintile | Quintile |
| Adoption (1/0) |  |  |  |  |  |
| Trained | 0.81 | 0.70 | 0.89 | 0.87 | 0.61 |
| Share (%) | (20.88) | 18.04 | (22.94) | (22.42) | (15.72) |
|  |  |  |  |  |  |
| Non-trained | 0.81 | 0.69 | 0.68 | 0.45 | 0.00 |
| Share (%) | (30.80) | 26.24 | (25.86) | (17.11) | (0.00) |
|  |  |  |  |  |  |
| Cowpea yield (kg/ha) |  |  |  |  |  |
| Trained | 171.63 | 402.74 | 781.43 | 1261.98 | 1727.17 |
| Share (%) | (3.95) | 9.27 | (17.98) | (29.04) | (39.75) |
|  |  |  |  |  |  |
| Non-trained | 61.17 | 420.05 | 785.67 | 1486.70 | 0.00 |
| Share (%) | (2.22) | 15.25 | (28.53) | (53.99) | (0.00) |
|  |  |  |  |  |  |
| Cowpea income (GHS) |  |  |  |  |  |
| Trained | 309.52 | 754.55 | 1208.07 | 1955.00 | 3396.73 |
| Share (%) | (4.06) | 9.90 | (15.85) | (25.64) | (44.55) |
|  |  |  |  |  |  |
| Non-trained | 359.44 | 679.42 | 1165.23 | 1967.19 | 3561.84 |
| Share (%) | (4.65) | (8.79) | (15.07) | (25.44) | (46.06) |

Note: Values in parentheses are in percentages. The row summation of the shares (percentages) adds up to 100. Exchange rate: 1 US$=GHS5.37 (Bank of Ghana, 2019).

*4.2 Stochastic dominance results*

The stochastic dominance analysis (SDA), highlighted in Figs. 3 and 4, is restricted to households matched based on their propensity score of participation whose distribution and region of common support ranges from 0.0195 to 0.9997. Households outside the region of common support were dropped. The good overlap between the density distribution of propensity scores for participants and non-participants of the CATP justifies the use of PSM and the basis for comparison using the SDA. Fig. 6 shows the cumulative distribution functions (CDFs) for cowpea yield and crop income for trained and untrained cowpea farmers. The results indicate that yield and crop income of participants stochastically dominate those of non-participants at yields below 1,000 kg/ha and crop income below GHS4,000 (US$745). However, for cowpea yields above 1,000kg/ha and crop income above GHS5,100 (US$950), the CDFs of both participants and non-participants are almost the same, but the crop income of non-participants stochastically dominate that of the participants for crop income ranging from GHS4,000 (US$745) to GHS5,100 (US$950). The nonparametric Kolmogorov–Smirnov test for first-order stochastic dominance also shows that the CDFs of participants stochastically dominate those of non-participants for cowpea yield and crop income at 1% level of significance. Based on the results, we conclude that conditional on observed characteristics, there is a higher probability that participants in a CATP will, on average, have higher yields and crop income than non-participants.

Fig. 3: Propensity score density distribution and common support region for adoption

Fig. 4: Propensity score density distribution and common support region for cowpea yield

Fig. 5: Propensity score density distribution and common support region for cowpea income

1. Cowpea yield (kg/ha) (b) Cowpea income (GHS)

Fig. 6: CDF of cowpea yield (a) and cowpea income (b) for trained and non-trained farmers

*4.3 Impact of training on welfare outcomes: ESR results*

Table 5 presents the full information maximum likelihood estimates of the determinants of participation in cowpea training (selection equation) and the impact of participation on adoption of climate-smart cowpea variety, cowpea yield, and crop income (outcome equations). The correlation coefficient (rho_2) in the adoption model is statistically significant while that of the yield and income model is not statistically significant, suggesting heterogenous results depending on the outcome variable. The exclusion restriction variable, access to a Member of Parliament or political figure, is statistically significant in the first stage model (participation in a CATP), but insignificant in the outcome equations, thus satisfying the instrument relevance condition. The positive coefficient confirms the expectation that access to a Member of Parliament or political figure increases the chance of being included in agricultural development projects. The falsification test established the admissibility of the instrument (Table A1).

Results from the selection equation indicate that participation in the CATP is significantly influenced by age, household size, membership in a FBO, membership in a VSLA, farm area, and access to a Member of Parliament or political figure. The age coefficient indicates that older household heads are more likely to participate in the CATP relative to younger household heads. Household size has a positive effect on participation in the CATP. Larger household size corresponds with higher food requirements. Thus, participation in the CATP equips the household head to make an informed decision that supports higher productivity and farm income. Membership in a FBO provides an opportunity for farmers to be part of development projects as most development organizations are more likely to work with farmer groups [4]. Several studies [33-35] have highlighted the critical role FBOs play in capacity building and adoption of agricultural technologies. Area under cowpea is associated with a higher probability of participation in the CATP. Farmers with a larger farm are more likely to experiment with new technologies gained from training on a portion of their farmland to verify the economic benefits. In addition, land serves as a proxy for wealth given that land is the main production asset for farmers [36-37]. Farmers with more land are expected to participate in agricultural training activities that provide information on modern inputs to enhance their yield relative to poorer cowpea producing households. Farmers who reside in Tolon, Savelugu, and Bawku Municipal districts are more willing to participate in cowpea training relative to farmers who resides in the Nadowli district. Consistent with the findings and reasons of [36], districts that are more likely to participate in cowpea training are located closely to national agricultural research systems and non-governmental organizations involved in research, training, and advisory services to smallholder farmers.

Table 5: Full information maximum likelihood results of the endogenous switching regression

| Variable | Adoption of climate-smart cowpea | | |  | Cowpea yield (kg/ha) | | |  | Cowpea income (GHS) | | |
| --- | --- | --- | --- | --- | --- | --- | --- | --- | --- | --- | --- |
|  | Selection | Non-trained | Trained |  | Selection | Non-trained | Trained |  | Selection | Non-trained | Trained |
| Sex | -0.152 | 0.047 | 0.062 |  | -0.293 | 201.581*** | 160.589* |  | -0.232 | 0.877*** | 0.544*** |
|  | (0.191) | (0.072) | (0.082) |  | (0.207) | (74.667) | (103.334) |  | (0.210) | (0.152) | (0.166) |
| Age | 0.036*** | -0.012*** | -0.013** |  | 0.031*** | -6.105 | 2.497 |  | 0.033*** | -0.027** | 0.004 |
|  | (0.011) | (0.004) | (0.006) |  | (0.011) | (4.959) | (5.699) |  | (0.011) | (0.011) | (0.010) |
| Education (years) | 0.020 | 0.005 | 0.009 |  | 0.026 | 10.889 | 6.096 |  | 0.027 | -0.021 | 0.012 |
|  | (0.018) | (0.007) | (0.007) |  | (0.019) | (7.464) | (9.182) |  | (0.020) | (0.016) | (0.015) |
| Household size | 0.043** | -0.001 | 0.009 |  | 0.055*** | -5.546 | 11.858 |  | 0.049** | 0.024 | 0.004 |
|  | (0.019) | (0.008) | (0.009) |  | (0.020) | (8.755) | (10.390) |  | (0.020) | (0.018) | (0.017) |
| Farming experience | -0.028*** | 0.012*** | 0.007 |  | -0.017 | 4.322 | -9.460* |  | -0.019 | 0.018* | 0.012 |
|  | (0.011) | (0.004) | (0.005) |  | (0.011) | (4.706) | (5.080) |  | (0.011) | (0.010) | (0.009) |
| Cooperative | 0.040 | -0.024 | -0.055 |  | -0.120 | 39.585 | 72.701 |  | -0.039 | -0.179 | 0.089 |
|  | (0.258) | (0.097) | (0.140) |  | (0.281) | (96.637) | (171.545) |  | (0.286) | (0.200) | (0.274) |
| FBO | 0.526*** | -0.031 | -0.091 |  | 0.536*** | -69.585 | -53.603 |  | 0.503*** | -0.122 | 0.069 |
|  | (0.161) | (0.064) | (0.092) |  | (0.177) | (74.965) | (101.777) |  | (0.178) | (0.156) | (0.170) |
| VLSA | 0.521*** | -0.011 | 0.063 |  | 0.580*** | 32.225 | -197.466* |  | 0.531*** | 0.162 | -0.025 |
|  | (0.182) | (0.073) | (0.097) |  | (0.196) | (84.367) | (115.378) |  | (0.202) | (0.175) | (0.180) |
| Area | 0.229* | -0.059 | 0.057 |  |  |  |  |  | 0.363*** | 0.021 | 0.193* |
|  | (0.123) | (0.052) | (0.056) |  |  |  |  |  | (0.132) | (0.134) | (0.115) |
| Tolon district | 0.253 | -0.683*** | -0.186 |  | 0.229 | 809.513*** | 463.185** |  | 0.661* | -0.597* | -0.815** |
|  | (0.344) | (0.135) | (0.169) |  | (0.357) | (124.650) | (200.674) |  | (0.385) | (0.310) | (0.358) |
| Savelugu district | 1.864*** | -0.192 | -0.153 |  | 1.924*** | 529.459*** | 736.831*** |  | 1.837*** | 0.404 | 0.472 |
|  | (0.370) | (0.127) | (0.226) |  | (0.343) | (181.840) | (229.182) |  | (0.351) | (0.411) | (0.401) |
| Yendi district | 0.279 | -0.517*** | -0.451*** |  | 0.376 | 35.353 | 87.541 |  | 0.443 | -0.255 | -0.458 |
|  | (0.350) | (0.117) | (0.150) |  | (0.331) | (114.007) | (188.611) |  | (0.331) | (0.247) | (0.308) |
| Bawku Municipal | 0.795** | -0.910*** | -0.695*** |  | 0.572 | 1022.657*** | 882.765*** |  | 0.758* | -0.414 | 0.250 |
|  | (0.396) | (0.140) | (0.180) |  | (0.374) | (135.844) | (205.780) |  | (0.391) | (0.304) | (0.341) |
| Binduri district | 0.329 | -0.594*** | -0.557*** |  | 0.333 | 977.737*** | 968.355*** |  | 0.548 | -0.560** | -0.195 |
|  | (0.352) | (0.117) | (0.150) |  | (0.325) | (107.666) | (185.651) |  | (0.335) | (0.247) | (0.309) |
| Wa West district | -0.120 | 0.081 | -0.064 |  | -0.188 | 53.198 | 400.819* |  | -0.232 | 0.067 | 0.498 |
|  | (0.340) | (0.120) | (0.172) |  | (0.350) | (121.812) | (217.357) |  | (0.211) | (0.251) | (0.345) |
| Access to Member of Parliament | 0.546** |  |  |  | 0.697*** |  |  |  | 0.556** |  |  |
|  | (0.216) |  |  |  | (0.226) |  |  |  | (0.231) |  |  |
| Distance extension | -0.002 |  |  |  | -0.007 |  |  |  | 0.000 |  |  |
|  | (0.011) |  |  |  | (0.012) |  |  |  | (0.012) |  |  |
| Constant | -3.386*** | 1.127*** | 1.299** |  | -3.210*** | 381.213* | 208.293 |  | -3.787*** | 7.248*** | 5.959*** |
|  | (0.581) | (0.216) | (0.568) |  | (0.565) | (221.573) | (488.655) |  | (0.608) | (0.545) | (1.027) |
| rho_1 |  | -0.189 |  |  |  |  | 0.293 |  |  |  | -0.171 |
|  |  | (0.539) |  |  |  |  | (0.323) |  |  |  | (0.454) |
| rho_2 |  |  | -0.960*** |  |  | -0.104 |  |  |  | -0.006 |  |
|  |  |  | (0.023) |  |  | (0.390) |  |  |  | (0.465) |  |
| *Model diagnostics* |  |  |  |  |  |  |  |  |  |  |  |
| Wald chi2 | 73.91*** |  |  |  | 92.30*** |  |  |  | 103.21*** |  |  |
| Log likelihood | -262.28 |  |  |  | -2546.54 |  |  |  | -533.96 |  |  |
| LR test $\chi^{2}(1)$ | 21.42*** |  |  |  | 0.77 |  |  |  | 0.14 |  |  |
| Observations | 319 |  |  |  | 320 |  |  |  | 320 |  |  |

***Notes***: Adoption of climate-smart cowpea is a dummy variable. Sigma is an auxiliary parameter (i.e., the square-root of the variance of the residuals of the endogenous switching regression model). Sigma (participants) associated with yield and income for participants are 0.0637 (0.055) and 410.991 (37.231), respectively, and sigma (non-participants) associated with yield and income for non-participants are 0.866 (0.042) and 423.438 (21.774), prespectively. Figures in parentheses are standard errors. Exchange rate: 1 US$=GHS5.37 (Bank of Ghana, 2019). *** p < 0.01 ** p < 0.05, and * p < 0.001.

Table 6 presents the estimated ATT of participation in cowpea training on the adoption of climate-smart cowpea variety, yield, and income. Compared to the results in Table 3, the ATT accounts for selection bias due to observable and non-observable characteristics between participants and non-participants. The results show that participating in the CATP significantly increases the adoption of climate-smart cowpea varieties and cowpea yield (ATT) by 75% and 119 kg/ha, respectively. The result shows that trained farmers record an estimated adoption rate of 78%, whereas the estimated adoption rate for trained farmers had they not participated (counterfactual adoption) in the training is just 3%. Participants in the CATP report an estimated yield of 888 kg/ha while the estimated yield for participants had they not participated (counterfactual yield) in the cowpea training is 770 kg/ha. The ATT’s value of 119 kg/ha represents a yield increase of 15%^[[4]](#footnote-4)^. The average cowpea yield of 888 kg/ha by participants under observed conditions is 63% of the average yield (1,400 kg/ha) and 36% of the potential yield (2,500 kg/ha) obtained from on-farm experiments [38]. For non-participants, the mean cowpea yield would have decreased by 107 kg/ha had they participated in the cowpea training. The expected cowpea income for participants is GHS1,470 (US$274) and the expected income of participants had they not participated (counterfactual income) in the cowpea training is GHS1,182 (US$220). The expected income for non-participants had they participated in the cowpea training is GHS1,350 (US$251) and the expected income for non-participants had they not participated in cowpea training is GHS1,096 (US$204). The ATT indicates that participating in cowpea training increases cowpea income by GHS289 (US$54), which represents an income gain of 24%. The results are consistent with the finding of previous studies by [36, 18, 39-40]. Based on the results, it can be concluded that the causal mechanism of higher welfare impacts of training is gained through high levels of adoption of improved cowpea varieties.

Concerning the adoption of climate-smart cowpea varieties, the results show that base heterogeneity is negative for both participants (BH_11_) and non-participants (BH_12_), while the transitional heterogeneity (TH_11_) is positive. The TH_11_ suggests that the effect of adoption of climate-smart varieties is more considerable for households that participated in the CATP relative to those that did not participate. Concerning cowpea yield, the effect of the base heterogeneity is positive for participants (BH_21_) and negative for non-participants (BH_22_). BH_21_ suggests that the effect of cowpea training is larger for participants than non-participants had they participated in the cowpea training. BH_22_ suggests that the cowpea yield effect is smaller for participants had they not participated in cowpea training relative to non-participants had they participated in cowpea training. In terms of cowpea income, we observed that the effect of base heterogeneity for participants (BH_31_) is greater than the base heterogeneity effect for non-participants (BH_32_) and is positive for both participants and non-participants. BH_31_ indicates that the effect of cowpea income is larger for participants had they not participated in cowpea training relative to non-participants had they participated in cowpea training. Similarly, BH_32_ indicates that the effect of cowpea income is higher for participants had they not participated in the CATP compared to non-participants had they participated in cowpea training. The transitional heterogeneity effect is positive for both yield and cowpea income. This implies that the effect on cowpea yield and income are larger for households that participated in the cowpea training relative to those that did not participate.

Table 6. Average expected cowpea income and land productivity by training status

| Sample | Training decision | | Treatment effect |
| --- | --- | --- | --- |
|  | To train | Not to train |  |
| *Adoption (1/0)* |  |  |  |
| Trained | 0.78 | 0.03 | ATT = 0.75 (0.04)*** |
| Non-trained | 0.85 | 0.62 | ATU = 0.23 (0.03)*** |
| Heterogeneity effects | BH_11_ = -0.07 | BH_12_ = -0.59 | TH_11_ = 0.52 |
| *Yield (kg/hectare)* |  |  |  |
| Trained | 888.21 | 769.59 | ATT = 118.62 (53.20)** |
| Non-trained | 719.57 | 826.45 | ATU = -106.88 (41.57)** |
| Heterogeneity effects | BH_21_ = 168.64 | BH_22_ = -56.86 | TH_21_ = 225.50 |
| *Crop income (GHS)* |  |  |  |
| Trained | 1470.40 | 1181.84 | ATT =288.56*** (110.85) |
| Non-trained | 1349.61 | 1096.08 | ATU =253.53***(74.35) |
| Heterogeneity effects | BH_31_ = 120.79 | BH_32_ = 85.76 | TH_31_ = 35.03 |

*Notes*: The numbers in parentheses associated with ATT and ATU are standard errors. Exchange rate: 1 US$=GHS5.37 (Bank of Ghana, 2019). *** p < 0.01, and ** p < 0.05.

*4.4 Robustness check*

The PSM and IPWRA estimates used as robustness checks for the ESR model are reported in Table 7. The results indicate that adoption of climate-smart cowpea varieties, cowpea yield, and crop income are significantly higher for participants compared to non-participants of the CATP. Participation in the CATP increased adoption, cowpea yield, and income increased by 17%, 175 kg/ha, and GHS410 (US$76.35), respectively, using the nearest neighbor matching method. Adoption of the climate-smart cowpea variety, cowpea yield, and income increased by 12%, 176 kg/ha, and GHS445 (US$83), respectively, for participants in the CATP when the kernel matching method is employed. The IPWRA estimates also show that participants in the CATP recorded a significantly higher climate-smart cowpea variety adoption, yield, and income than non-participants. Comparatively, the estimates of PSM and IPWRA are consistent with the estimates obtained from the ESR estimation. The results indicate that our findings are robust to different estimation techniques. Finally, we conducted a heterogeneous impact of cowpea training, which is necessary for designing effective and well-targeted training programs for rural farm households. The analysis is reported in appendix B.

Table 7: Propensity score matching and IPWRA estimate of cowpea training.

| Panel A | Adoption (1/0) | |  | Yield (kg/Hectare) | |  | Cowpea income (GHS) | |
| --- | --- | --- | --- | --- | --- | --- | --- | --- |
|  | ATT | Robust  Std. Error |  | ATT | Robust  Std. Error |  | ATT | Robust  Std. Err. |
| IPWRA | 0.165** | 0.082 |  | 128.40** | 61.44 |  | 551.36** | 242.15 |
|  |  |  |  |  |  |  |  |  |
| Panel B | | | | | | | | |
|  | Adoption (1/0) | |  | Yield (kg/Hectare) | |  | Cowpea income (GHS) | |
|  | ATT | Bootstrap  Std. Error |  | ATT | Bootstrap  Std. Error |  | ATT | Bootstrap  Std. Error |
| NNM | 0.165** | 0.077 |  | 174.86** | 87.66 |  | 410.30** | 206.18 |
| KNM | 0.121* | 0.073 |  | 175.92* | 97.26 |  | 445.22** | 213.42 |

Note: IPWRA is inverse probability weighted regression adjustment; NNM is nearest neighbor matching; and KNM is kernel matching. ** p < 0.05 and * p < 0.001.

**5. Conclusion and policy implications**

Agricultural training programs remain one of the mechanisms for introducing and disseminating modern technologies to farm households. Several studies have highlighted the role of training on household welfare based on farmer field schools and farmer-to-farmer extension delivery. In recent years, CATPs are gaining popularity. These training programs require participating farmers to gain informal certification after completing a set of practices – ranging from seed selection to postharvest management. However, there is limited evidence on the socio-economic impact of these intensive agricultural training programs. This paper makes an empirical contribution in addressing the paucity of information regarding the impact of intensive agricultural training capacity building in northern Ghana. Using farm household level data from the region and an ESR model, this study evaluates the impact of participation in cowpea training on the adoption of climate-smart cowpea varieties, yield, and cowpea income.

The results show that participation in the CATP is significantly influenced positively by age, household size, membership in a FBO, membership in a VSLA, farm area, access to a Member of Parliament, and access to a political figure. However, farming experience is negatively associated with participation in cowpea training. The result implies that future CATPs in northern Ghana must consider these factors in the selection process to ensure higher participation.

Results from the ESR model indicate that after accounting for both observed and unobserved heterogeneity, participation in the CATP is associated with a positive effect on the adoption of climate-smart cowpea varieties, yield, and cowpea income. Gains due to participation in cowpea training on good agronomic practices are 75% for the adoption of improved cowpea varieties, 118.26 kg/ha for cowpea yield, and GHS288.56 (US$49.88) for cowpea income.

The positive effect of cowpea training on welfare outcomes implies that continuous support and scaling-up of agricultural training programs in good agronomic practices and commercial orientation will enhance greater participation and impact on the welfare of farm households in Ghana. Agricultural development programs that enhance participation in CATPs must be promoted and sustained beyond the duration of commissioned projects or programs. Creating awareness and eliminating barriers to participation in CATPs can induce greater participation and enhance the adoption of improved varieties and welfare effects among farmers in northern Ghana.

**References**

[1] Fuglie, K., and Nin-Pratt, A. (2012). A Changing Global Harvest. 2012 Global Food Policy Report. Washington DC.

[2] Fuglie, K., Wang, S., And Ball, V. (2012). Productivity Growth in Agriculture: An International Perspective. London And Basingstoke.: CABI International.

[3] Martey, E., Kuwornu, J. K., & Adjebeng-Danquah, J. (2019). Estimating the effect of mineral fertilizer use on Land productivity and income: Evidence from Ghana. *Land Use Policy*, *85*, 463-475.

[4] [Asante-Addo, C.](https://www.emerald.com/insight/search?q=Collins%20Asante-Addo), [Mockshell, J.](https://www.emerald.com/insight/search?q=Jonathan%20Mockshell), [Zeller, M.](https://www.emerald.com/insight/search?q=Manfred%20Zeller), [Siddig, K.](https://www.emerald.com/insight/search?q=Khalid%20Siddig) and [Egyir, I.S.](https://www.emerald.com/insight/search?q=Irene%20S.%20Egyir) (2017), Agricultural credit provision: what really determines farmers’ participation and credit rationing?, [Agricultural Finance Review](https://www.emerald.com/insight/publication/issn/0002-1466)*,* Vol. 77 No. 2, pp. 239-256. <https://doi.org/10.1108/AFR-02-2016-0010>

[5] Anderson, J. R., & Feder, G. (2004). Agricultural extension: Good intention and hard realities. The World Bank Research Observer, 19(1), 41–60.

[6] [Feder, G.](https://www.emerald.com/insight/search?q=Gershon%20Feder), [Birner, R.](https://www.emerald.com/insight/search?q=Regina%20Birner) and [Anderson, J.R.](https://www.emerald.com/insight/search?q=Jock%20R.%20Anderson) (2011), "The private sector's role in agricultural extension systems: potential and limitations", [Journal of Agribusiness in Developing and Emerging Economies](https://www.emerald.com/insight/publication/issn/2044-0839), Vol. 1 No. 1, pp. 31-54. <https://doi.org/10.1108/20440831111131505>

[7] Godtland, E. M., Sadoulet, E., Janvry, A. de, Murgai, R., & Ortiz, O. (2004). The Impact of Farmer Field Schools on Knowledge and Productivity: A Study of Potato Farmers in the Peruvian Andes. *Economic Development and Cultural Change,* 53(1), 63–92*.* doi:10.1086/423253

[8] Davis, K., Nkonya, E., Kato, E., Mekonnen, D. A., Odendo, M., Miiro, R., & Nkuba, J. (2012). Impact of Farmer Field Schools on Agricultural Productivity and Poverty in East Africa. *World Development,* 40(2), 402–413. doi:10.1016/j.worlddev.2011.05.019

[9] Larsen, A. F., & Lilleør, H. B. (2014). Beyond the Field: The Impact of Farmer Field Schools on Food Security and Poverty Alleviation. *World Development,* 64, 843-859*.* doi:10.1016/j.worlddev.2014.07.003

[10] Nakano, Y., Tsusaka, T. W., Aida, T., & Pede, V. O. (2018). Is farmer-to-farmer extension effective? The impact of training on technology adoption and rice farming productivity in Tanzania. *World Development*, *105*, 336-351.

[11] Tambo, J. A., & Mockshell, J. (2018). Differential impacts of conservation agriculture technology options on household income in Sub-Saharan Africa. *Ecological economics*, *151*, 95-105. https://doi.org/ 10.1016/j.ecolecon.2018.05.005

[12] Yamane, T. (1967). Statistics: an introductory analysis (Second Edition). Book. New York: Harper and Row.

[13] Ministry of Food and Agriculture, MoFA. (2011). Agriculture in Ghana: *Facts and Figures* (p. 53), Accra, Ghana.

[14] Khonje, M., Manda, J., Alene, A.D. & Kassie, M. (2015). Analysis of adoption and impacts of improved maize varieties in eastern Zambia. *World Development*, *66*: 695–706.

[15] Becerril, J., & Abdulai, A. (2010). The impact of improved maize varieties on poverty in Mexico: A propensity score-matching approach. *World Development,* *38*(7): 1024–1035.

[16] Amare, M., Asfaw, S., & Shiferaw, B. (2012). Welfare impacts of maize–pigeonpea intensification in Tanzania. *Agricultural Economics, 43*(1): 27–43.

[17] Wooldridge, J.M. (2005). Instrumental estimation of the average treatment effect in the correlated random coefficient model. *Department of Economics,* Michigan State University, Michigan.

[18] Manda, J., Alene, A. D., Tufa, A. H., Abdoulaye, T., Wossen, T., Chikoye, D., & Manyong, V. (2019). The poverty impacts of improved cowpea varieties in Nigeria: A counterfactual analysis. *World Development*, *122*, 261-271

[19] Abdoulaye, T., Wossen, T., & Awotide, B. (2018). Impacts of improved maize varieties in Nigeria: Ex-post assessment of productivity and welfare outcomes. Food Security, 10(2), 369–379. <https://doi.org/10.1007/s12571-018-0772-9>.

[20] Tambo, A.J., Wunscher, T. (2014). More than adopters: the welfare impacts of farmer innovation in rural Ghana. Selected Paper Prepared for Presentation at the Agricultural & Applied Economics Association 2014 AAEA Annual Meeting.

[21] Asfaw, S., Shiferaw, B., Simtowe, F., & Lipper, L. (2012). Impact of modern agricultural technologies on smallholder welfare: Evidence from Tanzania and Ethiopia. *Food Policy*, *37*(3): 283–295.

[22] Di Falco, S., Veronesi, M., & Yesuf, M. (2011). Does adaptation to climate change provide food security? A micro-perspective from Ethiopia. *American Journal of Agricultural Economics,* *93*(3): 829–846.

[23] Shiferaw, B., Kassie, M., Jaleta, M., & Yirga, C. (2014). Adoption of improved wheat varieties and impacts on household food security in Ethiopia. Food Policy, 44, 272–284. https://doi.org/10.1016/j.foodpol.2013.09.012.

[24] Ricker-Gilbert, J., Jayne, T. S., & Chirwa, E. (2011). Subsidies and crowding out: A double-hurdle model of fertilizer demand in Malawi. *American journal of agricultural economics*, *93*(1), 26-42.

[25] Seck, A. (2017). Fertiliser subsidy and agricultural productivity in Senegal. *The World Economy*, *40*(9), 1989-2006.

[26] Ragasa, C., & Mazunda, J. (2018). The impact of agricultural extension services in the context of a heavily subsidized input system: The case of Malawi. *World development*, *105*, 25-47.

[27] Maddalla, G. S. (1983). Limited dependent and qualitative variables in econometrics. Cambridge, UK: Cambridge University Press.

[28] Di Falco, S., & Veronesi, M. (2013). How can African agriculture adapt to climate change? A counterfactual analysis from Ethiopia. *Land Economics*, *89*(4), 743-766.

[29] Lee, L. F., & Trost, R. P. (1978). Estimation of some limited dependent variable models with application to housing demand. *Journal of Econometrics*, *8*(3), 357-382.

[30] Lokshin, M., & Sajaia, Z. (2004). Maximum likelihood estimation of endogenous switching regression models. The Stata Journal, 4(3), 282–289.

[31] Mutenjea, M.J., Farnworthb, C.R., Stirlingc, C., Thierfeldera, C., Mupangwad, W., Nyagumboa, I. (2019). A cost-benefit analysis of climate-smart agriculture options in Southern Africa: Balancing gender and technology. Ecological Economics 163.126–137.

[32] Sheahan, M. & Barrett, C.B. (2017). Ten striking facts about agricultural input use in Sub-Saharan Africa. *Food Policy*, *67*: 12-25.

[33] Wossen, T., Abdoulaye, T., Alene, A., Haile, M. G., Feleke, S., Olanrewaju, A., & Manyong, V. (2017). Impacts of extension access and cooperative membership on technology adoption and household welfare. Journal of Rural Studies, 54, 223–233.

[34] Feleke, S., Manyong, V., Abdoulaye, T., & Alene, A. (2016). Assessing the impacts of cassava technology on poverty reduction in Africa. Studies in Agricultural Economics, 118, 101–111. <https://doi.org/10.7896/j.1612>.

[35] Ma, W., & Abdulai, A. (2016). Does cooperative membership improve household welfare? Evidence from apple farmers in China. Food Policy, 58, 94–102. https://

doi.org/10.1016/j.foodpol.2015.12.002.

[36] Tufa, A. H., Alene, A. D., Manda, J., Akinwale, M. G., Chikoye, D., Feleke, S., ... & Manyong, V. (2019). The productivity and income effects of adoption of improved soybean varieties and agronomic practices in Malawi. *World Development*, *124*, 104631.

[37] Abdulai, A., & Huffman, W. (2014). The adoption and impact of soil and water conservation technology: An endogenous switching regression application. Land Economics, 90(1), 26–43. <https://doi.org/10.1353/lde.2014.0009>.

[38] Ministry of Food and Agriculture, 2017. Planting for Food and Jobs Strategic Plan for Implementation (2017–2020). Ministry of Food and Agriculture, Accra, Ghana.

[39] Abdulai, A. N. (2016). Impact of conservation agriculture technology on household welfare in Zambia. *Agricultural Economics*, *47*(6), 729-741.

[40] Awotide, B. A., Alene, A. D., Abdoulaye, T., & Manyong, V. M. (2015). Impact of agricultural technology adoption on asset ownership: the case of improved cassava varieties in Nigeria. *Food Security*, *7*(6), 1239-1258.

**Appendices**

**Appendix A: Falsification test**

Table A1: Test of the validity of the instrument (falsification test)

|  | Cowpea training (dummy) | |  | Adoption of improved varieties (dummy) | |  | Cowpea yield (kg/ha) | |  | Cowpea income (GHS) | |
| --- | --- | --- | --- | --- | --- | --- | --- | --- | --- | --- | --- |
| Variable | Coefficient | Robust |  | Coefficient | Robust |  | Coefficient | Robust |  | Coefficient | Robust |
|  |  | Std. Error |  |  | Std. Error |  |  | Std. Error |  |  | Std. Error |
| Access to MP | 0.559** | 0.240 |  | 0.167 | 0.168 |  | 31.144 | 65.300 |  | -64.531 | 173.670 |
| Constant | -3.777*** | 0.552 |  | 0.631* | 0.149 |  | 838.234*** | 165.703 |  | 689.953 | 365.460 |
|  |  |  |  |  |  |  |  |  |  |  |  |
|  | LR chi2(1) = 5.43** | |  | F value =0.99 | |  | F value = 0.23 | |  | F value = 0.14 | |

Notes: *** p < 0.01 and ** p < 0.05.

**Appendix B: Heterogeneous treatment effects of cowpea training**

**Heterogeneous treatment effects**

*Heterogeneous treatment effects over propensity scores*

Fig. B1 shows the variation of ATT for yield and income scores over the estimated propensity scores. The results show that the ATT for yield and income varies significantly with the propensity. Moreover, the slope is negative for yield and positive for income. The positive slope suggests that the effect of the CATP on crop income is stronger for households with the highest propensity to participate in cowpea training. On the other hand, the negative slope suggests that the effect of training on cowpea yield is lower for households with the highest propensity to participate in cowpea training.

Fig. B1. Heterogeneity of treatment effects over propensity scores

*Heterogeneous effects of cowpea training over household characteristics*

Despite the positive impact of participation in the CATP on the welfare outcomes of rural households, we argue that welfare indicators may differ among different farm households. Understanding the heterogeneous impact of cowpea training is necessary for designing effective and well-targeted training programs for rural farm households. Following the approach by [33], we used the ATT of individual outcome variables (adoption, yield, and income) as a dependent variable in an OLS regression and ascertain how socio-economic characteristics explain variations in the estimated ATT. The results are reported in Table B1.

In terms of the adoption of climate-smart cowpea varieties, we find a statistically significant differential effect of the CATP among treated households with respect to age and residence in Savelugu, Yendi, Bawku Municipal, and Binduri districts. The results indicate that cowpea training exerts a lower effect for older farmers and farmers located in these districts. The estimated OLS results show that to a large extent, the CATP has heterogeneous effects given that membership in a VSLA, district dummies, and access to a political figure significantly influence the estimated ATT for cowpea yield among the treated farm households. The results indicate that being a member of a VSLA reduces the probability of benefiting from cowpea training. However, access to political figures and households who reside in Savelugu, Yendi, Bawku Municipal, Binduri, and Wa West districts benefit most from cowpea training. This suggests that increasing farmers’ access to agricultural training can maximize cowpea yield. With respect to cowpea income, we find a statistically significant differential effect of cowpea training among treated households with respect to sex, household size, farming experience, member of a VSLA, and member of a FBO. The results show that male-headed households and farmers who have several years of experience in cowpea farming benefit most from the CATP. This implies that identifying experienced cowpea farmers and using them as model farmers in technology dissemination may result in higher impacts on income.

Table B1: Heterogeneous treatment effects of cowpea training

| Variables | Adoption | Yield | Cowpea income |
| --- | --- | --- | --- |
| Sex | 0.036 | 73.893 | 976.246** |
|  | (0.139) | (155.538) | (445.241) |
| Age | -0.019*** | 5.385 | -20.989 |
|  | (0.006) | (8.448) | (16.764) |
| Education (years) | 0.004 | 1.846 | -42.047 |
|  | (0.013) | (14.384) | (37.943) |
| Household size | 0.006 | 9.764 | -69.925** |
|  | (0.011) | (14.759) | (34.837) |
| Farming experience | 0.009 | -10.256 | 25.093* |
|  | (0.006) | (6.892) | (13.970) |
| Cooperative | -0.230 | 14.330 | 860.122 |
|  | (0.210) | (291.200) | (844.646) |
| FBO membership | -0.121 | -44.601 | -639.602* |
|  | (0.123) | (156.540) | (333.970) |
| VLSA membership | -0.012 | -289.467* | -906.748** |
|  | (0.134) | (164.954) | (417.323) |
| Cowpea area | 0.063 | -149.139 | 90.824 |
|  | (0.090) | (89.752) | (245.969) |
| Tolon district | -0.507 | 152.321 | -389.858 |
|  | (0.327) | (366.088) | (873.060) |
| Savelugu district | -0.645*** | 991.714*** | -90.289 |
|  | (0.228) | (229.534) | (826.117) |
| Yendi district | -0.813*** | 100.919 | 269.482 |
|  | (0.230) | (278.174) | (895.259) |
| Bawku Municipal | -1.006*** | 889.147** | 810.725 |
|  | (0.257) | (340.825) | (882.979) |
| Binduri district | -1.172*** | 1,119.241*** | -85.629 |
|  | (0.255) | (281.870) | (972.452) |
| Wa West | -0.140 | 634.377** | 977.651 |
|  | (0.283) | (272.945) | (886.890) |
| Access/related to Member of Parliament | -0.038 | 444.682** | -245.270 |
|  | (0.160) | (173.193) | (502.179) |
| Distance to extension | 0.007 | -1.948 | 9.020 |
|  | (0.005) | (8.293) | (17.911) |
| Constant | 1.287*** | -302.315 | 1,128.166 |
|  | (0.389) | (518.898) | (1,089.606) |
|  |  |  |  |
| Observations | 109 | 109 | 109 |
| R-squared | 0.360 | 0.372 | 0.349 |

Note: The dependent variable is the ATT of each respective outcome indicators. Robust standard errors are in parentheses. *** p < 0.01, ** p < 0.05, and * p < 0.1.

1. The sampling frame consists of all cowpea producing districts in (former) northern Ghana consisting of Northern, Upper East, and Upper West regions. The zone is made up of 52 districts, with 26 in Northern, 15 in Upper East and 11 in Upper West regions. For more details on the top 10 cowpea producing districts, refer to the Ministry of Food and Agriculture (MoFA). (2017). Agriculture in Ghana Facts and Figures, 2016. Statistics, Research and Information Directorate (SRID) of MoFA, Accra.

   https://new-ndpc-static1.s3.amazonaws.com/CACHES/PUBLICATIONS/2016/04/16/AGRICULTURE-IN-GHANA-Facts+and+Figures-2010.pdf [↑](#footnote-ref-1)
2. The power of the test is the chance to reject the null hypothesis, given that the null hypothesis is false (i.e., given that the alternative hypothesis is true). It is the used to determine the sample size for a study. [↑](#footnote-ref-2)
3. A falsification test certifies the admissibility of the selection instrument as a valid instrument: If a variable is an appropriate selection instrument, it will affect the choice of participation in the CATP, but it will not affect the welfare or outcomes variables [28]. Our results indicate that the instrument can be considered as valid given that it is statistically significant in the participation in the CATP equation (equation X), but not significant in the outcome equations (equations Y and Z) (Table A1). [↑](#footnote-ref-3)
4. The percentage yield is computed as the ratio of the ATT yield (119 kg/ha) to the yield under the counterfactual scenario (770 kg/ha) and is expressed as a percentage. [↑](#footnote-ref-4)
